# Identifying the temporal electrophysiological and molecular changes that contribute to TSC-associated epileptogenesis

**DOI:** 10.1172/jci.insight.150120

**Published:** 2021-12-08

**Authors:** Linda M.C. Koene, Eva Niggl, Ilse Wallaard, Martina Proietti-Onori, Diana C. Rotaru, Ype Elgersma

**Affiliations:** Department of Clinical Genetics and Neuroscience, Erasmus MC, The ENCORE Expertise Center for Neurodevelopmental Disorders, Rotterdam, Netherlands.

**Keywords:** Neuroscience, Epilepsy, Ion channels, Transcription

## Abstract

Tuberous sclerosis complex (TSC), caused by heterozygous mutations in *TSC1* or *TSC2*, frequently results in intractable epilepsy. Here, we made use of an inducible *Tsc1*-knockout mouse model, allowing us to study electrophysiological and molecular changes of *Tsc1*-induced epileptogenesis over time. We recorded from pyramidal neurons in the hippocampus and somatosensory cortex (L2/L3) and combined this with an analysis of transcriptome changes during epileptogenesis. Deletion of *Tsc1* resulted in hippocampus-specific changes in excitability and adaptation, which emerged before seizure onset and progressed over time. All phenotypes were rescued after early treatment with rapamycin, an mTOR inhibitor. Later in epileptogenesis, we observed a hippocampal increase of excitation-to-inhibition ratio. These cellular changes were accompanied by dramatic transcriptional changes, especially after seizure onset. Most of these changes were rescued upon rapamycin treatment. Of the genes encoding ion channels or belonging to the Gene Ontology term action potential, 27 were differentially expressed just before seizure onset, suggesting a potential driving role in epileptogenesis. Our data highlight the complex changes driving epileptogenesis in TSC, including the changed expression of multiple ion channels. Our study emphasizes inhibition of the TSC/mTOR signaling pathway as a promising therapeutic approach to target epilepsy in patients with TSC.

## Introduction

Tuberous sclerosis complex (TSC) is a developmental disorder caused by an autosomal mutation in the *TSC1* or *TSC2* gene, with an estimated incidence of 1 in 6000 (1). Characteristic of the disorder is the development of tumor-like lesions in multiple organs, including the brain (2, 3). Patients with TSC show a variety of neurological problems like autism and epilepsy. Being often medically intractable and occurring early in life, epilepsy drastically lowers the quality of life of patients and their caretakers. Furthermore, the presence of early epilepsy correlates with the severity of autism and intellectual disability (4, 5). Hence, it is conceivable that treatment should start as soon as possible and preferably before seizures are detected to achieve the best outcome in terms of seizure control and neurological symptoms. The mechanism behind the development of epilepsy, a process called epileptogenesis, is currently unknown and needs to be understood to develop more efficient and early administrable pharmacological treatments (6).

The TSC complex is part of the mTOR pathway, where it inhibits the mTOR via inhibition of the small G protein called Ras homolog enriched in brain (RHEB). Loss-of-function mutations in the *TSC1* or *TSC2* gene result in increased levels of mTOR, dysregulating many cellular processes, including cell growth, autophagy, protein translation, and lipid and nucleotide synthesis (7). In neurons, mTOR also regulates ion channel expression and synapse formation as well as synapse function (8–12). How increased levels of mTOR drive these neuronal changes that ultimately lead to epilepsy remains largely unknown (13).

Previous studies used widely different experimental systems (e.g., cultured neurons versus brain slices, embryonic deletion versus induced adult deletion, sparse deletion versus global deletion) to determine how increased mTOR signaling changes neuronal circuits and thus leads to epilepsy (9, 12, 14–18). Although the effects of excessive mTOR signaling have been assessed on very different neuronal networks, a common finding was a change in intrinsic excitability (either hyper- and hypoexcitability) along with synaptic transmission in neurons that lack either *TSC1* or *TSC2*. Dysfunction in both excitability and/or synaptic transmission may underlie epilepsy (9, 10, 14), but the timeline of these changes regarding seizures remains unclear, and hence, to what extent the electrophysiological changes are driving the seizures or are the result of seizures.

To gain more insight into the process of mTOR-dependent epileptogenesis, we used a previously developed, brain-specific inducible *Tsc1* mouse model (*Tsc1*^fl/fl^-*Camk2a*-*Cre^ERT2^*), which shows spontaneous seizures 8 days after conditional deletion of the *Tsc1* gene in adult mice (19). Because seizure onset within this model is highly predictable, it is an ideal model to study mTOR-dependent epileptogenesis and to identify early cellular changes that eventually result in epilepsy.

Within this study, we used a whole-cell patch-clamp to study mTOR-dependent epileptogenesis in the hippocampus and somatosensory cortex (SSCx, L2/L3). In addition, we used RNA-Seq to identify differentially expressed genes throughout epileptogenesis in the hippocampus. This combination offers a unique approach to studying epileptogenesis over time with the potential to reveal the underlying mechanism of mTOR-dependent epileptogenesis. We demonstrate a robust increase of hippocampal excitability and impaired spike adaptation before seizure onset, which was fully rescued upon early and late rapamycin treatment. Furthermore, an increased excitation-to-inhibition ratio was observed in the hippocampus but only late in epileptogenesis. Last, by using RNA-Seq, we revealed significant changes in the expression of specific ion channels over time within the hippocampus, which are possibly driving the observed electrophysiological changes.

## Results

### Tsc1-Cre^+^ hippocampal neurons fire more action potentials with impaired adaptation ratio before seizure onset.

**Figure 1:**
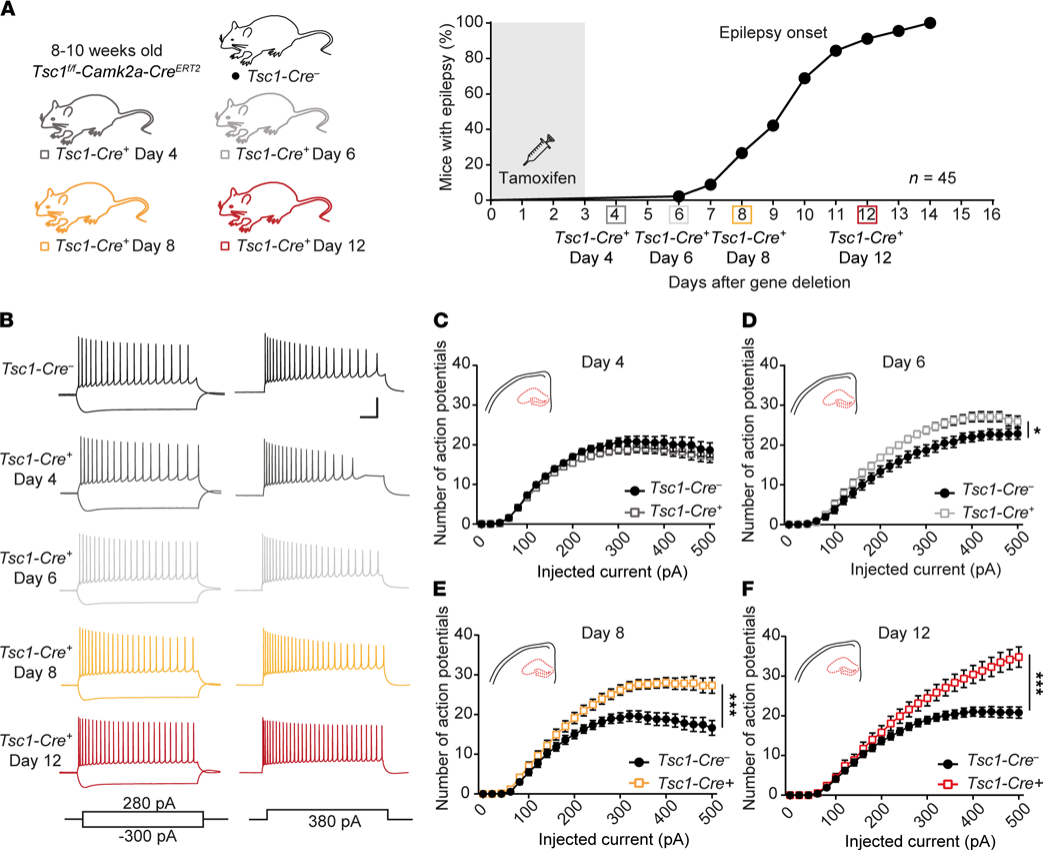
Increased action potential firing throughout epileptogenesis. (**A**) Conditional, Cre-dependent, homozygous *Tsc1* mouse model, color-coded for each experimental day (left) and timeline (right) of the experiments performed throughout epileptogenesis along with percentages of seizure onset on different days (based on previously published data; ref. 19). Squares on the timeline show the day of the patch-clamp measurements. (**B**) Representative examples of the whole-cell patch-clamp recordings from CA1 pyramidal neurons showing voltage responses to the current injection shown at the bottom. Scale bar indicates 100 ms and 40 mV. (**C**–**F**) Average number of action potentials of CA1 pyramidal neurons in response to increasing current injections of *Tsc1-Cre*^–^ and *Tsc1-Cre^+^* mice measured on day 4 (**C**), day 6 (**D**), day 8 (**E**), and day 12 (**F**) after gene deletion (day 4: *Tsc1-Cre*^–^ *n* = 22, 4 mice; *Tsc1-Cre^+^* *n* = 16, 3 mice; day 6: *Tsc1-Cre^–^* *n* = 16, 3 mice; *Tsc1-Cre^+^* *n* = 16, 3 mice; day 8: *Tsc1-Cre*^–^ *n* = 22, 5 mice; *Tsc1-Cre^+^* *n* = 22, 5 mice; day 12: *Tsc1-Cre*^–^ *n* = 21, 5 mice; *Tsc1-Cre^+^* *n* = 21, 5 mice; 2-way RM ANOVA).*P < 0.05, ***P < 0.001.

Previously, we showed that upon deletion of *Tsc1* in CAMK2A-positive neurons, mice start to have epilepsy after 10 ± 2 days (ref. 19 and Figure 1A). To gain more insight into the cellular changes, we measured intrinsic cell properties over time using whole-cell patch-clamp in CA1 pyramidal neurons. We started measuring neuronal excitability on day 4 after gene deletion. At this time point, *Tsc1-Cre^+^* neurons behaved similarly to control neurons (Figure 1, B and C). Interestingly, only 2 days later (day 6), a significant increase in the number of action potentials was observed during constant-current injections in *Tsc1-Cre^+^* neurons (2-way repeated measures [RM] ANOVA: *F*_1, 30_ = 7.1; *P* = 0.01; Figure 1, B and D), which became much stronger at 8 days after gene deletion (Figure 1, B and E), a time point when the first mice showed seizures (2-way RM ANOVA: *F*_1, 42_ = 17.3; *P* = 0.0002). Because the number of epileptic seizures increased over time (19), we tested if the excitability of neurons at day 12 further increased compared with day 8. Indeed, the observed increase in the number of action potentials progressed over time, showing even more action potentials on day 12 (2-way RM ANOVA: *F*_1, 40_ = 8.7; *P* = 0.005; Figure 1, B and F).

**Figure 2:**
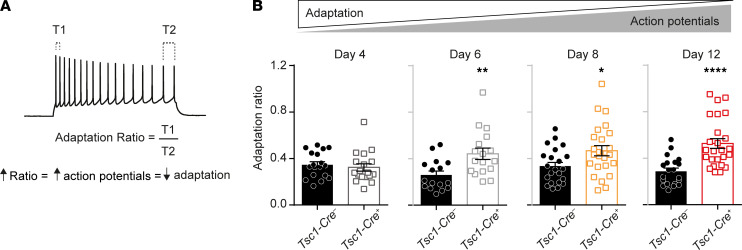
Increased adaptation ratio reflecting less adaptation in TSC neurons progressing throughout epileptogenesis. (**A**) Calculation of the adaptation ratio dividing the time (ms) between the first 2 fired action potentials and last 2 action potentials at sweeps where 16–18 action potentials were fired. (**B**) Averaged adaptation ratios throughout epileptogenesis (day 4: *Tsc1-Cre*^–^ *n* = 18, 3 mice; *Tsc1-Cre^+^* *n* = 18, 3 mice; day 6: *Tsc1-Cre*^–^ *n* = 16, 3 mice; *Tsc1-Cre^+^* *n* = 17, 3 mice; day 8: *Tsc1-Cre*^–^ *n* = 22, 3 mice; *Tsc1-Cre^+^* *n* = 25, 3 mice; day 12: *Tsc1-Cre*^–^ *n* = 20, 3 mice; *Tsc1-Cre^+^* *n* = 25, 3 mice). A 1-way ANOVA and Dunnett’s post hoc was used with *Tsc1-Cre^–^* as control. Error bars indicate SEM. *P < 0.05, **P < 0.01, ****P < 0.0001.

To get insight into the different classes of ion channels that may contribute to the increased hyperexcitability phenotype, the first action potential was analyzed at rheobase of all data sets that were significantly changed (day 6, day 8, and day 12). This analysis did not reveal significant changes in active or passive membrane properties, except for an increased capacitance and half-width on day 12 (Supplemental Table 1; supplemental material available online with this article; https://doi.org/10.1172/jci.insight.150120DS1). Additionally, adaptation ratios were calculated by dividing the time the time (T, in ms) between the first 2 action potentials (T1) by the time between the 2 last action potentials (T2) at the current step, producing 16–18 action potentials (Figure 2A). If neurons show a high adaptation ratio, i.e., a T1/T2 value close to 1, they adapt less and fire more action potentials. A low adaptation ratio, i.e., T1/T2 more toward 0, indicates that neurons adapt more. Since *Tsc1-Cre^+^* neurons fire more action potentials, an increased adaptation ratio is expected, reflecting less adaptation. Indeed, neurons of *Tsc1-Cre^+^* mice showed increased adaptation ratios. This increased adaptation ratio was significant on day 6 and progressed on day 8 and day 12 (day 4: T_33_ = 1.3, *P* = 0.19; day 6: T_31_ = 3.1, *P* = 0.004; day 8: T_44_ = 2.2, *P* = 0.04; day 12: T_43_ = 4.5, *P* < 0.0001; Figure 2B). Taken together, 6 days after *Tsc1* gene deletion, *Tsc1-Cre^+^* CA1 pyramidal neurons start to fire more action potentials and show an increased adaptation ratio, which progresses over time.

### Early and late in vivo rapamycin treatment rescues increased hippocampal excitability and soma size.

**Figure 3:**
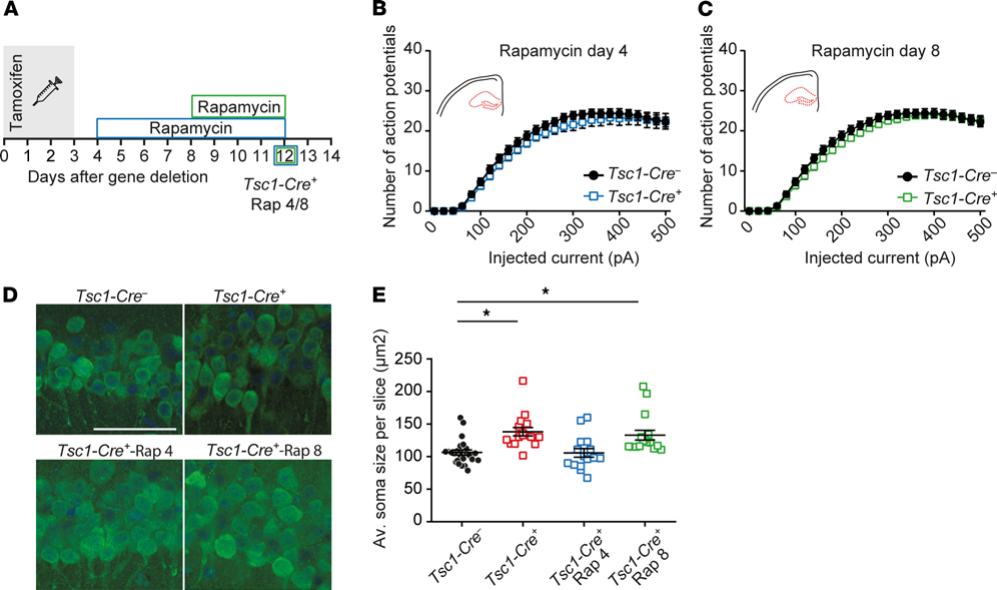
In vivo rapamycin treatment rescues hippocampal hyperexcitability and soma size. (**A**) Experimental timeline of rapamycin treatments (5 mg/kg) where blue illustrates early treatment starting immediately, on day 4 (1 day after the last tamoxifen injection), and green indicates treatment starting on day 8. The squares indicate the day of patch-clamp measurements. (**B** and **C**) Average number of action potentials of pyramidal CA1 neurons after increasing current injections of *Tsc1-Cre*^–^ and *Tsc1-Cre^+^* mice measured on day 12 with rapamycin treatment starting on day 4 (*Tsc1-Cre*^–^ *n* = 30, 4 mice; *Tsc1-Cre^+^* -Rap 4 *n* = 26, 4 mice) and day 8 (*Tsc1-Cre^+^* -Rap 8 *n* = 26, 4 mice; 2-way RM ANOVA). (**D**) Representative confocal pictures with a NeuN and DAPI staining of CA1 pyramidal neurons of *Tsc1-Cre*^–^ and *Tsc1-Cre^+^* day 12, *Tsc1-Cre^+^*-Rap 4, and *Tsc1-Cre^+^*-Rap 8 mice. Scale bar indicates 50 μm. (**E**) Average soma size per slice (15–20 cells per slice) per group (black: *Tsc1-Cre*^–^: *n* = 24, 5 mice; red: *Tsc1-Cre^+^* day 12: *n* = 16, 4 mice; blue: *Tsc1-Cre^+^* -Rap 4: *n* = 15, 3 mice, and green: *Tsc1-Cre^+^* -Rap 8: *n* = 16, 4 mice; 1-way ANOVA; Dunnett’s with *Tsc1-Cre^–^* as control). Error bars indicate SEM. *P < 0.05.

Previously we showed that early treatment of *Tsc1-Cre*^+^ mice (day 4) with 5 mg/kg rapamycin prevents seizure development (19). To assess whether the increased amount of action potentials in CA1 pyramidal neurons is mTOR dependent and reversible after epilepsy has started, we measured excitability in 2 rapamycin treatment conditions. The first cohort of mice received rapamycin treatment starting on day 4, before the observed increase in CA1 excitability and epilepsy (*Tsc1-Cre^+^*-Rap 4). We expected that this cohort would not develop seizures and would not show changes in excitability. The second cohort received rapamycin on day 8, a time point at which there was significantly increased excitability and epilepsy started to appear (*Tsc1-Cre^+^*-Rap 8; Figure 3A). Because the excitability phenotype was the strongest on day 12, we performed our measurements at this time point. As expected, neurons of the first cohort of *Tsc1-Cre^+^*-Rap 4 mice showed the same firing pattern as control *Tsc1-Cre*^–^ neurons (Figure 3B). Interestingly, neurons of the second cohort, *Tsc1-Cre^+^*-Rap 8 mice, showed a full rescue as well (Figure 3C). These results indicate that the hyperexcitability observed in *Tsc1-Cre*^+^ mice is mTOR dependent, reversible, and possibly responsible for epileptic seizures.

Similar to other studies (20–23), we previously showed increased soma size at day 12 after *Tsc1* gene deletion (19). To test whether this phenomenon can be rescued by rapamycin treatment, we performed a NeuN staining to label neurons on slices of day 12 *Tsc1-Cre*^–^ and *Tsc1-Cre*^+^, *Tsc1-Cre*^+^-Rap 4, and *Tsc1-Cre^+^*-Rap 8 mice. In line with previous experiments, *Tsc1-Cre*^+^ CA1 pyramidal neurons were significantly larger (Figure 3, D and E) whereas neurons of *Tsc1-Cre^+^*-Rap 4 mice were not different from control *Tsc1-Cre*^–^ neurons. Interestingly, rapamycin treatment on day 8 (*Tsc1-Cre^+^*-Rap 8) did not rescue the soma size, while it did rescue the hyperexcitability, suggesting that these 2 changes are independent from each other (1-way ANOVA: *F*_3, 67_ = 8.3, *P* < 0.0001; Dunnett’s: *Tsc1-Cre*^–^ vs. *Tsc1-Cre^+^* *P* = 0.0006; *Tsc1-Cre^–^* vs. *Tsc1-Cre^+^*-Rap 4 *P* = 0.99; *Tsc1-Cre*^–^ vs. *Tsc1-Cre^+^*-Rap 8 *P* = 0.0043). In line with the soma size data, cell capacitance was rescued only by early rapamycin treatment (Supplemental Figure 1). The finding that brief rapamycin treatment reversed excitability but not soma size suggests that increased soma size is not causally related to the changes in excitability.

### Loss of TSC results in increased soma size but does not affect intrinsic firing properties of cortical L2/L3 pyramidal neurons.

**Figure 4:**
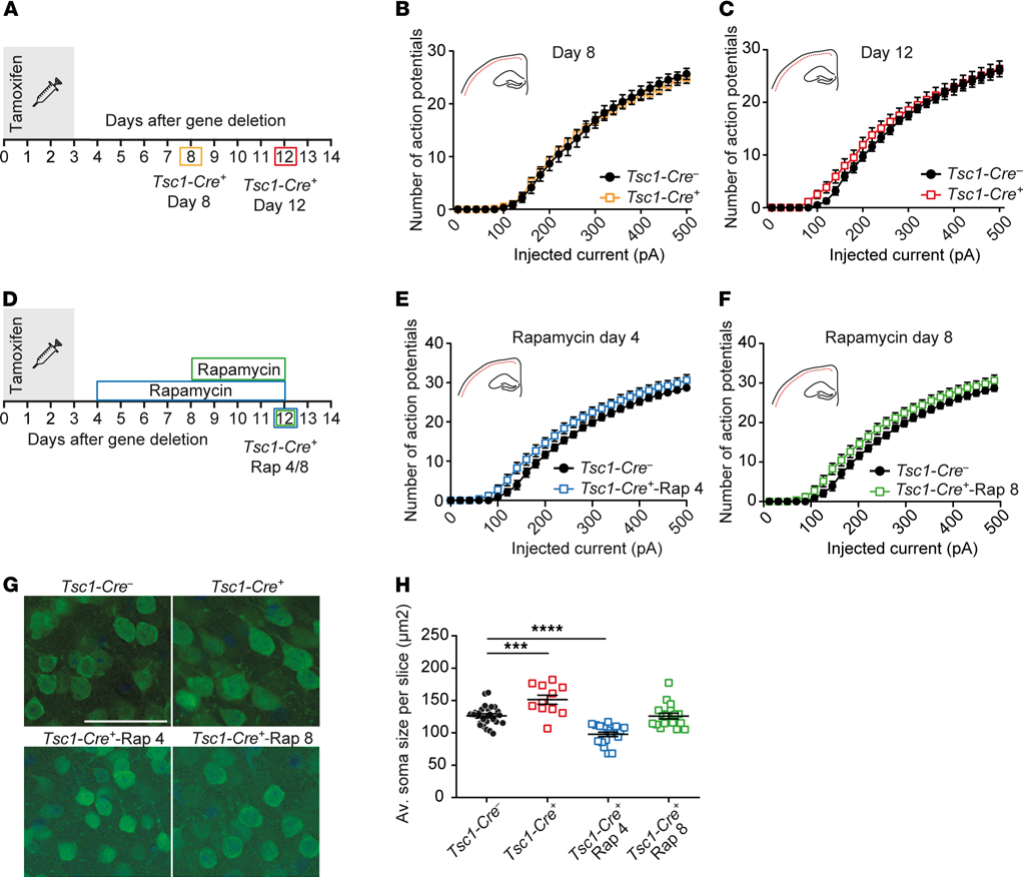
Normal intrinsic firing properties during epileptogenesis but increased mTOR-dependent soma size increase in SSCx L2/L3 pyramidal neurons. (**A**) Timeline of the patch-clamp experiments. The colored squares indicate the day of patch-clamp measurements. (**B** and **C**) Average number of action potentials of SSCx L2/L3 pyramidal neurons after increasing current injections of *Tsc1-Cre^–^* and *Tsc1-Cre^+^* mice measured on day 8 (**B**: *Tsc1-Cre^–^* *n* = 16, 4 mice; *Tsc1-Cre^+^* *n* = 19, 5 mice) and day 12 after gene deletion (**C**: *Tsc1-Cre^–^* *n* = 22, 4 mice; *Tsc1-Cre^+^* *n* = 23, 3 mice; 2-way RM ANOVA; Dunnett’s with *Tsc1-Cre^–^* as control). (**D**) Experimental timeline of the patch-clamp experiments and fluorescence staining for NeuN (green) and DAPI (blue). The squares indicate the day of patch-clamp measurements. (**E** and **F**) The average number of action potentials of L2/L3 pyramidal neurons in the SSCx after increasing current injections of *Tsc1-Cre^–^* and *Tsc1-Cre^+^* mice measured on day 12 with rapamycin treatment starting on day 4 (*Tsc1-Cre^–^* *n* = 13, 4 mice; *Tsc1-Cre^+^*-Rap 4 *n* = 11, 4 mice) and day 8 (*Tsc1-Cre^+^*-Rap 8 *n* = 15, 4 mice; 2-way RM ANOVA; Dunnett’s with *Tsc1-Cre^–^* as control). (**G**) Representative confocal pictures with a NeuN (green) and DAPI (blue) staining of SSCx L2/L3 pyramidal neurons of *Tsc1-Cre^–^*, *Tsc1-Cre^+^* day 12, *Tsc1-Cre^–^*-Rap 4, and *Tsc1-Cre^+^*-Rap 8 mice. Scale bar demonstrates 50 μm. (**H**) Average soma size SSCx L2/L3 pyramidal neurons per slice (15–20 cells per slice) per group (black: *Tsc1-Cre^–^*: *n* = 24, 5 mice; red: *Tsc1-Cre^+^* day 12: *n* = 11, 4 mice; blue: *Tsc1-Cre^+^*-Rap 4: *n* = 19, 3 mice, and green: *Tsc1-Cre^+^*-Rap 8: *n* = 18, 4 mice; 1-way ANOVA; Dunnett’s with *Tsc1-Cre^–^* as control). Error bars indicate SEM. ***P < 0.001, ****P < 0.0001.

Next, we asked whether similar changes as observed in CA1 neurons were also present in the SSCx. Therefore, we recorded L2/L3 pyramidal neurons of the SSCx. Because *Tsc1* is deleted in all CAMK2A-positive neurons (19), we hypothesized that similarly to CA1 pyramidal neurons, *Tsc1-Cre^+^* neurons in the SSCx would fire more action potentials, which would be reversible by rapamycin treatment. In contrast with our expectations, excitability was unaffected in L2/L3 pyramidal neurons of the SSCx 8 days after gene deletion as well as 12 days after gene deletion (Figure 4, A–F). However, we did observe an increase of soma size in L2/3 neurons 12 days after deletion, which, in contrast to the hippocampus, was rescued upon rapamycin treatment that started on day 8 and resulted in smaller soma sizes after treatment with rapamycin from day 4 (1-way ANOVA: *F*_3, 37_ = 26, *P* < 0.0001; Dunnett’s: *Tsc1-Cre*^–^ vs. *Tsc1-Cre^+^* *P* = 0.0001; *Tsc1-Cre*^–^ vs. *Tsc1-Cre^+^*-Rap 4 *P* < 0.0001; *Tsc1-Cre*^–^ vs. *Tsc1-Cre^+^*-Rap 8 *P* = 0.15; Figure 4, G and H). These data indicate that increased mTOR levels affect neurons differently in the hippocampus compared with the SSCx. In addition, these data further support the notion that increased soma size is not mechanistically related to neuronal excitability.

### Hippocampal synaptic imbalance is only present in the epileptic state.

**Figure 5:**
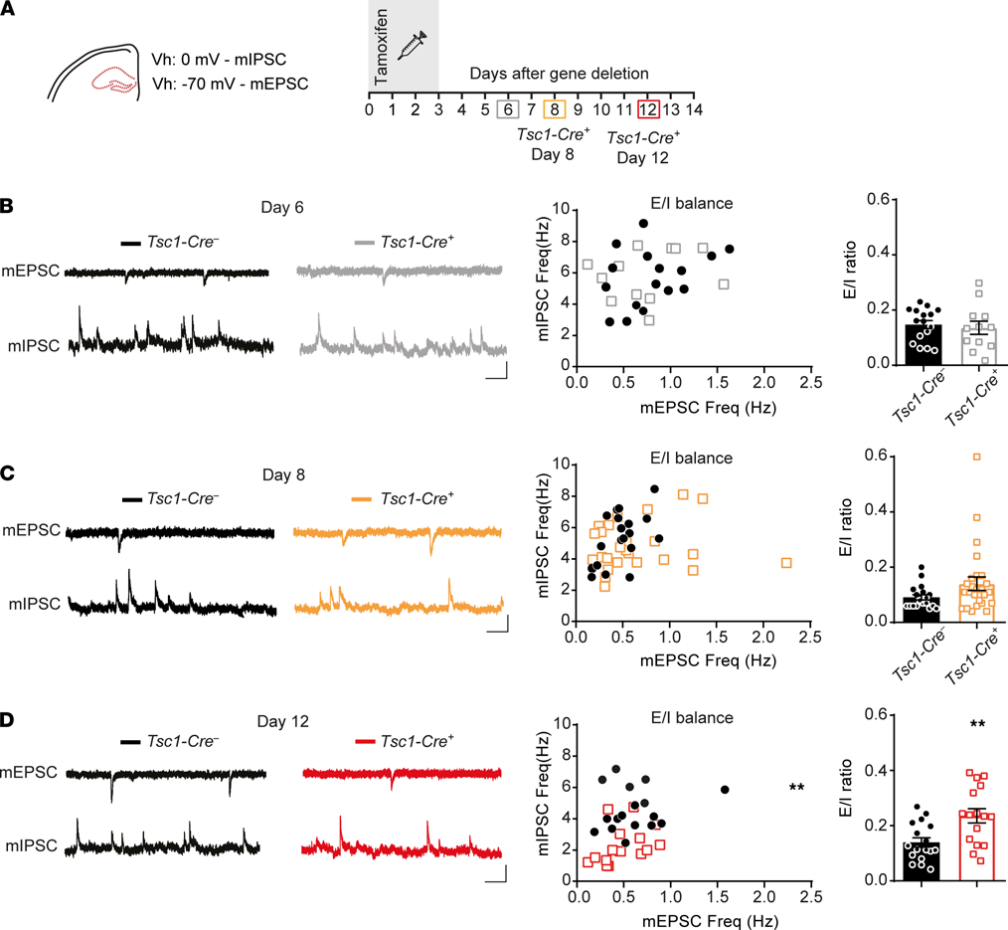
Hippocampal synaptic imbalance in late epileptogenesis. (**A**) Experimental timeline of the synaptic transmission measurements of CA1 pyramidal neurons. The colored squares indicate the day of patch-clamp measurements. Miniature inhibitory postsynaptic currents (mIPSCs) were recorded on a holding potential (Vh) of 0 mV and miniature excitatory postsynaptic currents (mEPSCs) at –70 mV in the presence of bath-applied tamoxifen. (**B**–**D**) Representative traces of mEPSCs and mIPSCs of *Tsc1-Cre*^–^ and *Tsc1-Cre^+^* mice (left) and scatter plots of averaged mIPSC and mEPSC frequencies for each cell (middle). The mEPSC/mIPSC frequency ratio of the same cells is depicted in the bar graph (right). In **B**, day 6 (*Tsc1-Cre*^–^ *n* = 16, 3 mice; *Tsc1-Cre^+^* *n* = 12, 3 mice; independent *t* test), **C**, day 8 (*Tsc1-Cre*^–^ *n* = 26, 4 mice; *Tsc1-Cre^+^* *n* = 30, 6 mice; independent *t* test) and **D**, day 12 (*Tsc1-Cre*^–^ *n* = 17, 4 mice; *Tsc1-Cre^+^* *n* = 16, 3 mice; independent *t* test). Scale bars indicate 100 ms and 20 pA. Error bars indicate SEM. **P < 0.01.

Previous studies reported increased excitatory transmission, decreased inhibitory transmission, or a combination of the two, leading to an overall increased excitation-to-inhibition ratio and therefore increased firing of the neuronal network (9, 10). Conversely, changes in excitability can lead to homeostatic changes in synaptic transmission (24). To study whether there are changes in the excitation/inhibition ratio (E/I) and if so, whether these changes precede or follow the changes in excitability, we measured miniature inhibitory postsynaptic currents (mIPSCs) and miniature excitatory postsynaptic currents (mEPSCs) in the hippocampus (Figure 5A). Both mIPSCs and mEPSCs were recorded within the same cell to calculate an E/I ratio for each cell. Additionally, to study action potential–dependent events, spontaneous EPSCs and IPSCs (sEPSCs and sIPSCs) were measured on day 8.

Both mIPSCs’ and mEPSCs’ frequencies were not different from control neurons on day 6 after gene deletion, the day on which excitability differences appeared (Figure 5B; Supplemental Table 2). On day 8 after gene deletion, a small, insignificant increase of mEPSCs’ and normal sEPSCs’ frequencies (Figure 5C and Supplemental Figure 2) was observed. Moreover, an insignificant decrease in mIPSCs’ and sIPSCs’ frequencies were observed in *Tsc1-Cre^+^* neurons. Together, the small differences in mEPSC and mIPSC did not yield a significant difference in E/I ratio (T_26_ = 0.4, *P* = 0.7). In contrast, a significant reduction in mIPSCs was observed 12 days after gene deletion, resulting in a significantly higher E/I ratio (T_31_ = 3.2, *P* = 0.003; Figure 5D). Event amplitude ratios appeared normal at all measured time points (Supplemental Figure 3). Looking into event kinetics regardless of the ratios, mIPSCs recorded at day 12 showed significantly smaller amplitudes and slower rise times (Supplemental Table 2). mEPSCs recorded on day 6 showed a significantly slower rise time, but on day 12 significantly smaller amplitudes with faster decay times were observed (Supplemental Table 2). Overall, these data indicate that changes in synaptic transmission are secondary to intrinsic cellular changes. Moreover, because these synaptic changes do not precede the seizure onset, it is unlikely that they are a driver of epileptogenesis.

**Figure 6:**
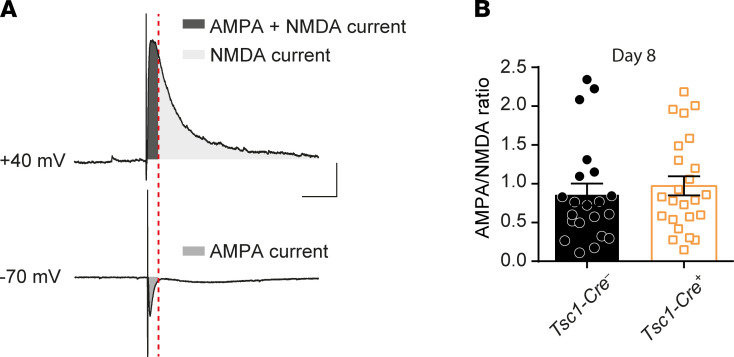
AMPA/NMDA ratio is unaffected in *Tsc1-Cre^+^* day 8 mice. (**A**) Method of AMPA- and NMDA-dependent current isolation for each cell by extracellular stimulation. To isolate the NMDA current, the first part of the +40 mV recorded current (AMPA + NMDA current; dark gray) was left out. (**B**) The calculated average AMPA/NMDA ratio of *Tsc1-Cre^–^* and *Tsc1-Cre^+^* recorded on day 8 after gene deletion is shown (*Tsc1-Cre^–^* *n* = 16, 3 mice; *Tsc1-Cre^+^* *n* = 12, 3 mice; independent *t* test). Scale bars indicate 100 ms and 100 pA. Error bars indicate SEM.

Previous reports showed an NMDA receptor subunit composition change resulting in increased currents in TSC mouse and patient cells (11). Since our mEPSC recordings mostly reflect currents dependent on α-amino-3-hydroxy-5-methyl-4-isoxazolepropionate (AMPA) (25), we looked more specifically at NMDA currents by recording NMDA/AMPA ratio in CA1 hippocampal pyramidal neurons by triggering EPSCs with extracellular stimulation. Neurons were voltage-clamped at –70 mV to record AMPA-dependent currents and were clamped on +40 mV to record NMDA within the same cell (Figure 6A). No differences in the NMDA/AMPA ratio were found comparing *Tsc1-Cre^+^* day 8 CA1 pyramidal neurons to *Tsc1-Cre*^–^ neurons (Figure 6B; *Tsc1-Cre*^–^ [*n* = 21; 4 mice] mean: 0.86 ± 0.14 vs. *Tsc1-Cre^+^* [*n* = 24; 4 mice] mean: 0.97 ± 0.12; T_43_ = 0.58, *P* = 0.56).

### Tsc1 deletion induces major changes in gene expression, which are rescued with rapamycin treatment.

**Figure 7:**
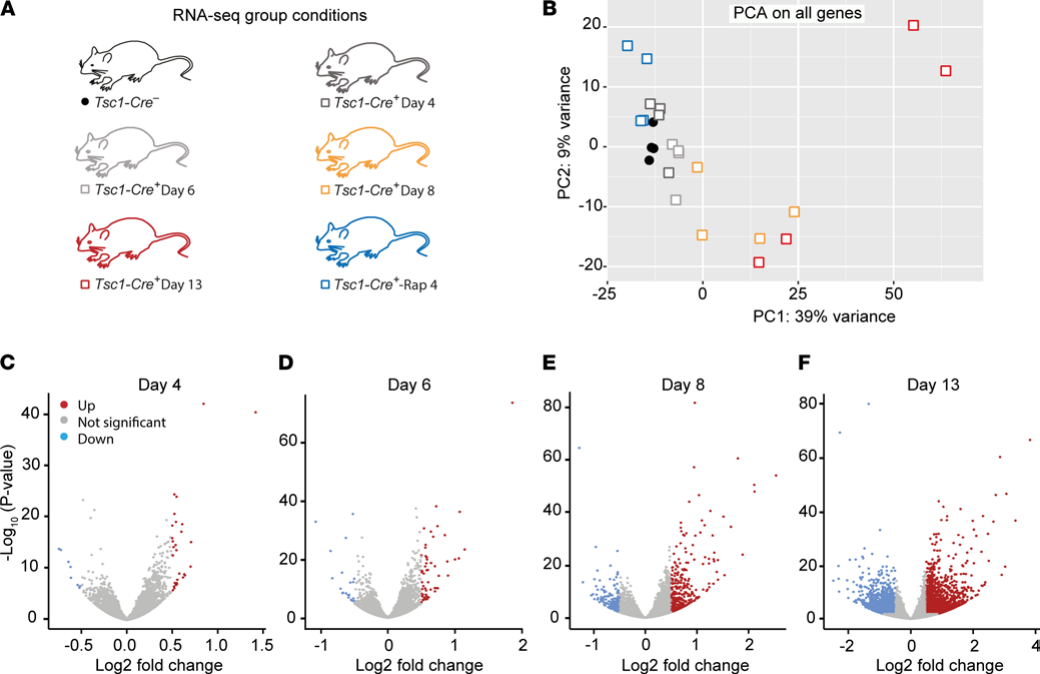
mTOR-dependent epileptogenesis affects the hippocampal transcriptome. (**A**) Experimental group conditions of the RNA-Seq samples. All groups included 4 mice. (**B**) Principal component analysis (PCA) plot of all mice included in the RNA-Seq analysis showing the variation between and within conditional groups. (**C**–**F**) Volcano plots showing the significance of upregulated (red) and downregulated (blue) genes of *Tsc1-Cre^+^* day 4, *Tsc1-Cre^+^* day 6, *Tsc1-Cre^+^* day 8, and *Tsc1-Cre^+^* day 13 mice compared with *Tsc1-Cre*^–^ mice.

To gain more insight into the molecular mechanisms underlying *Tsc1*-induced epilepsy, RNA-Seq transcriptome analysis was performed on hippocampal brain samples. RNA was extracted at multiple time points after gene deletion (day 4, day 6, day 8, and day 13) to follow the time course of epileptogenesis. To identify transcripts that are under the direct control of mTOR signaling (i.e., affected by *Tsc1* deletion and rescued by administering rapamycin), hippocampi of early rapamycin-treated mice (10 mg/kg; *Tsc1-Cre^+^* -Rap 4) were included in the data set as well (Figure 7A).

A principal component analysis (PCA) of this data set showed that, as time after *Tsc1* gene deletion increased, the samples shifted to the right along the PC1 axis, the component on which the samples showed the largest differences. This indicates that the samples became more different from *Tsc1-Cre*^–^ control mice over time. On the axis of the second most important component (PC2), we observed a downward shift of the samples over time, with the notable exception of samples taken on day 13, which showed a very large variation. This variation may reflect the large difference in seizure load between *Tsc1-Cre^+^* day 13 animals (ref. 19 and Figure 7B). Importantly, *Tsc1-Cre^+^* day 4 and day 6 samples clustered close to *Tsc1-Cre*^–^ samples, demonstrating similar expression profiles among these groups. Moreover, day 13 *Tsc1-Cre^+^* animals treated with rapamycin clustered close to the *Tsc1-Cre*^–^ mice, indicating that RNA expression of these mice was similar to control animals despite the deletion of the *Tsc1* gene.

To visualize the number of differentially expressed (DE) genes [*P* < 0.05; log(fold change) > 0.5 or < –0.5] in *Tsc1-Cre^+^* mice throughout epileptogenesis, volcano plots were generated for every time point (Figure 7, C–F). Over time, the number of DE genes increased, with a peak on day 13 (genes up: 1936; down: 1709). On day 4, ±4 days before seizure onset, 29 genes were significantly upregulated and 8 genes downregulated, whereas on day 6, 56 and 20 genes were significantly up- and downregulated, respectively. On day 8, an important time point identified as seizure onset, 392 genes were significantly upregulated and 243 downregulated. This means that from day 4 to day 6 the number of DE genes almost doubled, while from day 6 (when mice were not epileptic yet) to day 8 (the start of seizure onset), the number of DE genes was 7-fold increased. In addition, from the start of seizure onset (day 8) to a full epileptic state (day 13), approximately 5 times more DE genes were observed.

### Fifty-six genes are substantially dysregulated before or around seizure onset.

**Figure 8:**
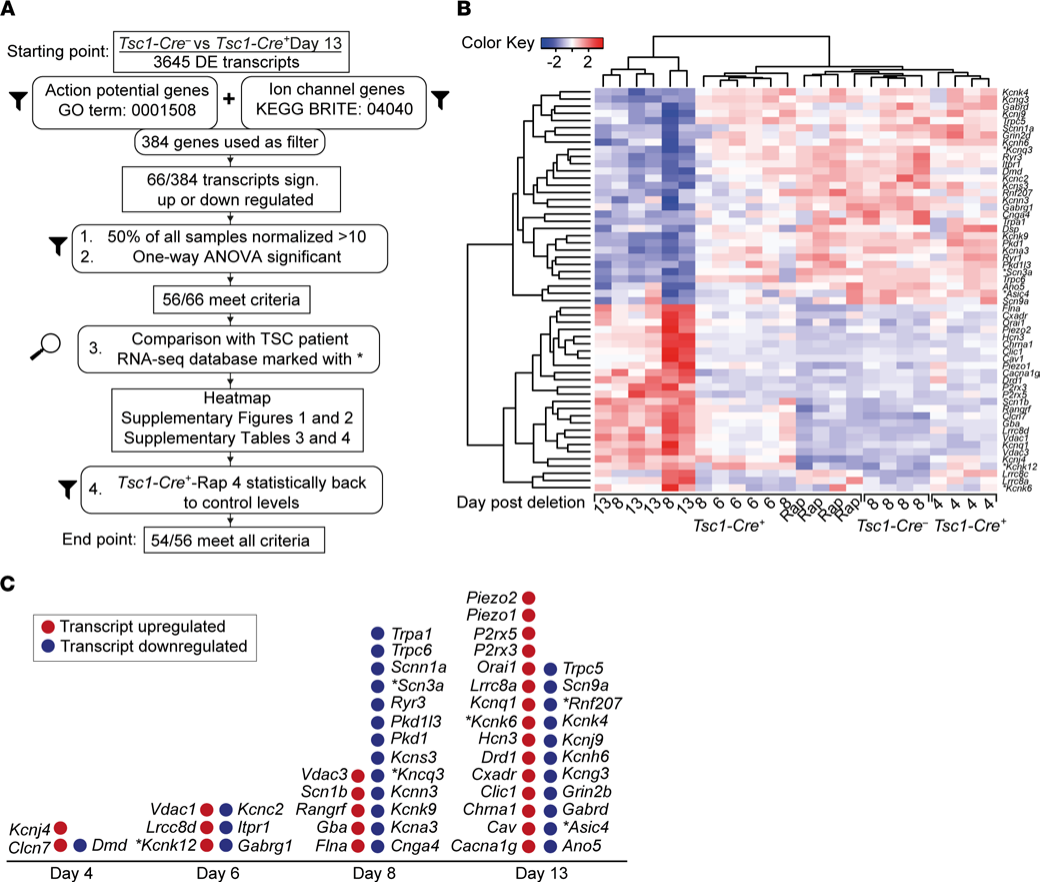
Fifty-four DE ion channel and/or action potential–related transcriptomes throughout epileptogenesis rescued upon rapamycin treatment. (**A**) Flowchart of the analysis used based on the KEGG BRITE 04040 and GO term action potential (GO: 0001508) gene lists and additional inclusion criteria used as a selection of genes of interest. At the start, 3645 DE transcripts from *Tsc1-Cre^+^* day 13 mice versus *Tsc1-Cre*^–^ mice were used to end up with 56 DE transcripts of which 54 were rescued with rapamycin treatment. (**B**) Heatmap depicting the *z* scores of the 56 genes identified (GO term 0001508 action potential) that are significantly up- or downregulated (indicated as red or blue) in *Tsc1-Cre^–^* and *Tsc1-Cre^+^* day 4, *Tsc1-Cre^+^* day 6, *Tsc1-Cre^+^* day 13, and *Tsc1-Cre^+^*-Rap 4 mice. (**C**) The 54 significant and rescued-upon-rapamycin genes of interest were plotted over time. If the gene was also found in the TSC patient RNA-Seq database, the gene is highlighted with an asterisk.

To identify candidate transcripts that could potentially underlie the observed hyperexcitability and the adaptation ratio impairment that preceded the seizures, we focused on genes DE between *Tsc1-Cre*^–^ and *Tsc1-Cre^+^* day 13. Since we were looking for potential target genes that affect action potential firing, we selected genes encoding ion channels (Kyoto Encyclopedia of Genes and Genomes [KEGG] BRITE: 04040, 308 genes) and genes of the Gene Ontology (GO) term action potential (GO: 0001508), resulting in a curated list of 384 unique genes (Figure 8A). Of these genes, 56 transcripts were significantly different in a 1-way ANOVA when directly comparing expression levels across conditions (Supplemental Tables 3 and 4). Expression levels of these 56 genes over time, including the group of mice treated with rapamycin, are displayed (*z* scores) in an unsupervised, clustered heatmap (Figure 8B). Within this heatmap, *Tsc1-Cre^–^* and *Tsc1-Cre^+^*-Rap 4 samples clustered together, indicating similar expression patterns of rapamycin-treated and control samples. In addition, (most) *Tsc1-Cre^+^* day 8 samples clustered with *Tsc1-Cre^+^* day 13, probably reflecting transcriptome changes induced by seizures. Since rapamycin treatment rescues the excitability phenotype and epilepsy, we examined whether the 56 transcripts would be rescued by rapamycin treatment as well. Strikingly, 54 of the 56 transcripts showed a full rescue, and expression levels were indistinguishable from *Tsc1-Cre^–^* (Supplemental Tables 3 and 4 and Supplemental Figures 4 and 5).

Plotting the normalized counts of individual genes at all time points and the rapamycin condition gives us an additional view on expression levels over time. Notably, approximately half of the genes were downregulated during epileptogenesis (Supplemental Figure 4 and Supplemental Table 3) and the other half upregulated (Supplemental Figure 5 and Supplemental Table 4). In addition, half of the genes were already significantly dysregulated (Supplemental Tables 3 and 4) before or around seizure onset, indicating that these genes could potentially be driver genes for epileptogenesis. Consistent with that, most genes showed the biggest change on day 8 and day 13, the time points with the largest hyperexcitability phenotypes (Figure 8C).

## Discussion

Using our previously designed TSC mouse model, we identified distinct electrophysiological and major transcriptome changes that appeared in the early phases of mTOR-dependent epileptogenesis. Moreover, our mouse model allowed us to follow these changes along the different stages of the epileptogenic process. Through a combination of whole-cell patch-clamp recordings and RNA-Seq, we uncovered brain region–specific changes and time-dependent effects of mTOR increase. Thus, in the hippocampus but not in the cortex from *Tsc1-Cre^+^* mice, we observed an increase in firing frequency with impaired spike adaptation starting before seizure onset and progressing over time. Later during epileptogenesis, we observed a decrease in inhibitory transmission in the hippocampus along with increased soma size in both hippocampal and cortical neurons of *Tsc1-Cre^+^* mice. Our analysis revealed dramatic time-dependent changes of the transcriptome. This included transcripts encoding multiple potassium channels, along with a few calcium and sodium channels. This suggests that mTOR-dependent epileptogenesis induces expression changes of multiple channels. Importantly, our electrophysiological and molecular phenotypes could both be rescued by rapamycin treatment.

By inducing sparse *Tsc1* deletion in the CA1, it was previously shown that intrinsic excitability was decreased and synaptic driven excitability unaffected (9). The authors reported that the decreased intrinsic firing reflects lower membrane resistance and higher capacitance. This is in contrast with our data obtained in CA1 upon widespread *Tsc1* deletion. An explanation for these contradictory results is the different mouse models used. In both models, increased levels of mTOR and increased soma sizes were observed, but mTOR activation might be required in multiple cells within the network to trigger a change in ion channel expression and cause increased excitability. In line with this, it was recently shown that expression of active RHEB in a subset of neurons can induce hyperexcitability in distally connected neurons that do not express active RHEB (18). In addition, there appear to be notable brain region–specific effects of *Tsc1* deletion, as we showed that, in contrast to hippocampal CA1 neurons, L2/L3 pyramidal neurons showed no observable electrophysiological changes in our model. Similarly, *Tsc1* deletion in the striatum caused hyperexcitability in striatonigral neurons but not in the striatopallidal neurons (26), highlighting the different roles of mTOR in neuronal subtypes *and* brain regions. Finally, the effects of mTOR activation can be age dependent. Increased levels of mTOR could interfere with brain maturation in young mice, causing cellular changes that are not present upon mTOR activation in adult mice (27).

Our findings support previous data suggesting not only that mTOR has specific effects on different brain regions but also that the degree of TSC1 loss at the network level matters, influencing the functioning of individual neurons (28–31). In line with our results, sparse *Tsc1* deletion in the hippocampus is associated with a decrease in inhibition while the excitatory transmission is unaffected (9). We found that the synaptic changes occurred rather late in epileptogenesis, which could indicate that the synaptic changes may be a secondary effect, i.e., driven by the seizures, or may require a prolonged time to develop. A previous study of epileptogenesis in a rat model also indicates that expression changes of synaptic genes are probably secondary to seizures (32). Importantly, these findings follow our previous data showing few effects of treatments aiming at increasing neuronal inhibition (19).

We found that deletion of *Tsc1* resulted in 3645 DE genes when measured 13 days after gene deletion. Upon filtering transcripts that encode ion channels or belong to the GO term action potential, we identified 56 DE genes that met our statistical criteria. We found that 27 (50%) of these DE genes were already significantly DE (Supplemental Tables 3 and 4) before or just around seizure onset, indicating that these genes could encode proteins ultimately responsible for epileptogenesis. Unlike the electrophysiological changes caused by severe channelopathies in rare monogenic epilepsy syndromes (33), the hyperexcitability phenotype present in our TSC mouse model may result from small cumulative changes, not in one but in several ion channels. In line with this notion, we observed an increase of DE genes over time, which parallels a progressive increase over time of neuronal hyperexcitability and seizure frequency (19). Moreover, most gene transcripts did not reach a statistically significant difference until day 8 while the hyperexcitability phenotype appeared already at day 6. However, our analysis has statistical limitations; we do not know the relationship between protein expression levels and mRNA transcripts, nor do we know the relationship between protein levels and the electrophysiological changes. Our data are in line with previous studies describing multiple changes in hippocampal gene expression during the epileptogenic process (32, 34–37). Importantly, we observed that almost all transcriptional changes were reversed by rapamycin treatment, supporting that excessive mTOR signaling is the main dysfunction leading to epilepsy.

Around seizure onset, changes in a subset of transcripts would likely favor a hypoexcitability phenotype (*Cnga4*, *Pkd1*, *Kcnc2*, *Dmd*, *Kcnj4*, *Kcnk12*, *Flna*), but the majority of changes would favor a hyperexcitability phenotype (*Gabrg1*, *Kcns3*, *Kcna3*, *Kcnn3*, *Kcnq3*, *Scnn1a*, *Itpr1*, *Gba*, *Scn1b*, *Kcnk9*, *Vdac1*). Notably, many of these changes involve potassium channels, well-known for their role in regulating both the active and passive levels of membrane voltages (38). It is important to mention that different from voltage-gated sodium and calcium channels, potassium channels are thought to have diversified in evolution to maintain neuronal excitation within limits (38, 39). Thus, the combined decreased expression of *Kcna3* (Kv1.3), *Kcnk9* (TASK3), *Kcnn3* (SK3), *Kcng3* (Kv7.3), and *Kcns3* (Kv9.3) around day 8 could lead to increased chances of persistent membrane depolarization, which ultimately may lead to epilepsy. For example, the decreased Kv9.3 levels may lead to a decreased conduction of the Kv2.1 (*Kcnb1*; refs. 40, 41), a well-known voltage-gated channel that regulates firing frequency (42). In addition, we found a trend of reduced expression of the calcium-conducting ryanodine receptor *Ryr1* on day 8 and a significant reduction of *Ryr3*, which are both located around the cluster formations of the Kv2.1 in the neuronal plasma membrane (40). Our findings of significant changes in multiple genes related to Kv2.1 functionality could indicate a change in its behavior without changing the expression of the Kv2.1 channel itself.

Besides transcript levels of several potassium channels, transcripts of voltage-gated sodium channels *Scn3a* (Nav1.3 α subunit) and *Scn1b* (Nav1.1 β subunit) were found to be affected as well. The Nav1.3 channel has been linked to neurodevelopmental disorders, brain malformation, and childhood-onset, treatment-resistant epilepsy (43–46). Notably, this channel is also significantly lower expressed in cortical tuber tissue from patients with TSC, highlighting the clinical relevance of our findings (47). *SCN1B* is another gene frequently found related to early-onset epilepsy and even linked to sudden unexpected death in epilepsy (48, 49). Sudden death is also observed in our mouse model (19). Interacting with both voltage-gated sodium and potassium channels, this β subunit has many functions in neurons, influencing voltage and conductive behavior of sodium and potassium channels. For this reason, the *Scn1b* subunit could play a significant role in mTOR-related epileptogenesis.

Several non–ion channel genes belonging to the action potential GO term were changed before and around the start of seizures, including *Dmd*, *Flna*, *Gba*, and *Rangrf*. The transcript *Dmd* encoding dystrophin was significantly increased (*P* = 0.02) starting at day 4 before changes in excitability were observed. Since dystrophin has been strongly associated with epilepsy in patients with Duchenne muscular dystrophy (50–52), it might be important in the epileptogenic process. *Flna* encodes filamin A, an actin cross-linking protein in which a loss-of-function mutation is associated with neuronal heterotopia and epilepsy (53). In our mouse model, levels of *Flna* went up after *Tsc1* deletion and were fully rescued after rapamycin treatment. This finding is in line with previous reports where increased levels of FLNA were directly related to mTOR activity in a mouse model of TSC and focal cortical dysplasia type II with epilepsy (54, 55). FLNA may lead to hyperexcitability by regulating the insertion of ion channels within the membrane (56, 57). *Gba* encodes RAN guanine nucleotide release factor and is found to interact with the Nav1.5 channel (*SCNA5*) in cardiac muscle cells and neurons. Through this mechanism, *Gba* could also influence neuronal excitably and thereby contribute to epileptogenesis (58).

The high sensitivity of RNA-Seq analysis allowed us to pick up subtle differences pointing toward a cumulative effect of mTOR-dependent changed ion channel expression. However, how this translates exactly to protein changes at the synapse or cell membrane remains unknown and would be difficult to establish due to the lack of sensitivity in Western blot and proteomic approaches. Although transcript levels are a poor predictor of protein abundance, a high predictive value was reported for membrane-associated proteins (like ion channels; ref. 59).

In line with previous studies (19), we observed increased soma sizes in *Tsc1-Cre^+^* neurons in both the hippocampus and the cortex; however, we observed an important difference between the 2 regions. The increased soma size observed in the SSCx could be rescued upon late rapamycin treatment and even resulted in smaller soma sizes when administered early. In contrast, increased hippocampal soma size could only be rescued by rapamycin upon early treatment. This could indicate that the rate of TSC1 decrease in the cortex has different kinetics compared with the hippocampus, but we previously reported (19) that this does not appear to be the case. Another explanation for this region-specific difference could be that mTOR fulfills different functions in different neuronal subtypes. This would be in line with the patch-clamp experiments showing no differences at any time point throughout epileptogenesis in the cortex, while it did result in early hippocampal changes. Furthermore, rapamycin rescued this phenotype in the hippocampus but did not affect the cortical neurons, suggesting a fundamental mTOR-related difference between brain regions. In addition, a recent study looking into neuronal subtypes of human epileptic and nonepileptic cortices concluded that some neuronal subtypes are more affected by epileptogenesis than others. For instance, neurons in L2/L3 SSCx could be less susceptible to epileptogenic changes than the hippocampal neurons (34).

We showed that targeted mTOR inhibition by rapamycin prevented or reversed hippocampal hyperexcitability upon early and late rapamycin treatment, respectively. Moreover, early intervention with rapamycin prevented widespread transcriptome changes. Given the major changes in gene expression that were observed upon seizure onset, our results further highlight the importance of treating epilepsy as specifically and early as possible to limit the damage induced by the seizures themselves (4, 6). Besides treatment with rapamycin (sirolimus) or its analog everolimus, other mTOR inhibitors could be exploited as well. Unfortunately, known mTOR pathway–specific inhibitors, including AZD5088 (dual mTOR) and PF4708671 (S6 kinase), are not as effective as rapamycin in inhibiting seizures in our mouse model (19). Potentially, a small molecule inhibitor that targets the small GTPase RHEB1 (60) could be a suitable candidate, since RHEB is the direct downstream target of TSC. Moreover, since we previously have shown that RHEB1 protein levels are the rate-limiting step in the TSC/mTOR pathway (61, 62) and the deletion of *Rheb1* can prevent seizures in the inducible *Tsc1* model (19), the activity of the pathway could potentially be reduced by antisense oligonucleotide–mediated targeting of *Rheb1* mRNA.

## Methods

### Mice

For patch-clamp experiments, male and female adult (8–10 weeks old) conditional *Tsc1*^fl/fl^-*Camk2*a-Cre^ERT2^ knockout mice (*Tsc1^tm1Djk^*: MGI: 2656240 and *Tg(Camk2a-cre/ERT2)2Gsc*: MGI: 3759305) were used as described previously (19). For the RNA-Seq experiments, hippocampal tissue was collected from 16- to 22-week-old male and female mice from the same mouse model. Mice were kept under a 12-hour light/12-hour dark cycle with ad libitum access to food and water. All mice received an i.p. injection of tamoxifen 20 mg/mL for 4 days, resulting in *Tsc1* deletion in CAMK2A neurons after tamoxifen injections in Cre^+^ carrying mice, hereafter called *Tsc1-Cre*^+^ mice: *Tsc1^fl/fl^-Tg(Camk2a-Cre^ERT2+^)*. Similar to Cre^+^ mice, littermates without Cre — *Tsc1^fl/fl^-Tg(Camk2a-CreERT*^–^*)* — received 4 tamoxifen injections and were used as controls (hereafter called *Tsc1-Cre*^–^). The experimenter checked the welfare of the epileptic mice daily.

### Slice preparation and electrophysiology

Coronal slices of 300 μm containing both dorsal hippocampus and SSCx were prepared at days 4, 6, 8, and 12 after gene deletion. The slicing solution contained (in mM) C_5_H_14_ClNO 110, KCl 2.5, NaH_2_PO_4_ 1.2, NaHCO_3_ 26, d-glucose 25, CaCl_2_ 0.5, and MgSO_4_ 10, while being saturated with 95% O_2_/5% CO_2_. After cutting, the slices were incubated at ±34°C for 5 minutes in cutting solution and then transferred to recording solution containing (in mM) NaCl 125, KCl 3, NaH_2_PO_4_ 1.25, NaHCO_3_ 26, d-glucose 10, CaCl_2_ 2 and MgSO_4_ 1 for another 15 minutes. Next, the slices were left at room temperature for an hour before the experiments.

Somatic whole-cell patch-clamp recordings were obtained from visually identified dorsal CA1 or layer 3 SSCx pyramidal neurons with an upright microscope using infrared-differential interference contrast optics (BX51WI, Olympus). All recordings were done at 30°C ± 1°C. All recordings were performed as previously described (18, 63). Series resistance was not corrected but monitored throughout the experiment. Only recordings with a stable series resistance lower than 20 MΩ were included. Membrane potentials were not corrected for liquid junction potential. Resting membrane potential was measured immediately after the break-in. The internal solution (pH of 7.2–7.4 adjusted with KOH and osmolarity of 280 ± 3) contained (in mM): K-gluconate 125, NaCl 10, HEPES 10, EGTA 0.2, MgATP 4.5, NaGTP 0.3, and Na-phosphocreatine 10 for the current clamp experiments. For voltage-clamp experiments it contained (in mM) cesium gluconate 125, CsCl 5, NaCl 8, HEPES 10, EGTA 1, K-phosphocreatine 2, MgATP 2, and NaGTP 0.3. For the sIPSC experiments, we used a high-chlorine intracellular solution, which contained (in mM) K-gluconate 77, KCl 77, HEPES 10, EGTA 0.2, MgATP 4.5, NaGTP 0.3, and Na-phosphocreatine 10.

### Recording protocols and analysis

#### Current clamp.

Action potentials were triggered from a holding potential of –70 mV by 750 ms square step current starting at –300 pA with increments of 20 pA. Action potentials were analyzed using Clampfit 10.7.0.3 (Molecular Devices). For each cell, the number of action potentials per sweep was counted, and the properties of the first action potential at rheobase were analyzed. The parameters taken into account were: maximum rise time, maximum decay time, amplitude, half-width, and threshold. The threshold was calculated by plotting the first derivative of the trace. The threshold was defined when the first derivative was lower than 10 mV/ms. Adaptation ratios were calculated by dividing the time between the first 2 action potentials (T1) and the last 2 action potentials (T2) of the sweep where 16–18 action potentials were observed. The cells that did not reach that number were left out of this analysis (see figure legends for *n*).

#### Voltage-clamp.

Action potential–dependent sEPSCs and sIPSCs were recorded as previously described (64) in the absence of tamoxifen (TTX). sEPSCs were recorded in the presence of 10 μM bicuculine and sIPSCs in the presence of 10 μM cyanquixaline (CNQX). mEPSCs and mIPSCs of the same cell were recorded in the presence of 1 μM TTX. The analysis of mEPSC and mIPSC frequency and properties, as well as the ratios, were calculated for each cell as previously described (64). At least 50 events were manually selected to analyze further event kinetics. Recordings with fewer events were excluded from further analysis.

To measure the AMPA-NMDA currents, glutamatergic inputs in striatum radiatum were stimulated in the presence of bicuculine (10 μM) using an extracellular double-well capillary glass electrode. First, the AMPA current was triggered while holding the cell at –70 mV, for 10 repetitions. Second, the holding potential was set at +40 mV to record NMDA current for another 10 repetitions. From the recordings, an average of 10 sweeps was taken for each cell. To obtain the NMDA current, the peak amplitude at +40 mV was calculated at a time point when the AMPA current decayed to more than 10% of its peak amplitude.

### Drugs

For some experiments, mice received a daily i.p. injection with 5 or 10 mg/kg rapamycin (LC Laboratories) on day 4 after gene deletion (*Tsc1-Cre^+^*-Rap 4) or on day 8 after gene deletion (*Tsc1-Cre^+^*-Rap 8). Doses were based on previous studies from our lab (19). TTX and bicuculine were obtained from Invitrogen. To record sIPSCs, 10 μM CNQX was used to block AMPA currents.

### Immunohistochemistry and soma size measurement

After the patch-clamp experiments, slices were fixated overnight in 4% paraformaldehyde at 4°C. Slices were stained according to the previously published methods (19, 65). In short, slices were preincubated in a 10% normal horse serum and 0.5% of Triton X-100 solution (MilliporeSigma T8787). The primary antibody was incubated overnight (NeuN 1:1000 anti-rabbit; MilliporeSigma, ABN78). The secondary antibody was applied for 3 hours at room temperature (1:200 anti-rabbit, A488; Thermo Fisher Scientific, AB_143165), followed by a DAPI staining (1:10.000; Invitrogen, D3571). Pictures were taken with a Zeiss confocal microscope at 40× original magnification. Soma sizes were measured using Zen software (version 7.0; 2011).

### RNA isolation

RNA was extracted from *Tsc1-Cre^+^* mice at 4 different time points during epileptogenesis: day 4, day 6, day 8, and day 13. RNA was extracted from *Tsc1-Cre*^–^ mice on day 8 after tamoxifen injection and *Tsc1-Cre*^+^ treated with 10 mg/kg rapamycin on day 13 (*Tsc1-Cre^+^*-Rap). Mice were anesthetized with isoflurane before decapitation. The hippocampi were isolated and immediately homogenized in TRIzol (15596-026, Invitrogen). Total RNA was isolated using the PureLink RNA Mini Kit (12183018A, Thermo Fisher Scientific) according to the manufacturer’s instructions.

### RNA-Seq library preparation and sequencing

Library preparation and sequencing were completed at Philips (Eindhoven, the Netherlands). Stranded total RNA libraries were prepared using the Illumina TrueSeq Stranded Total RNA LT kit (with Ribo-Zero Gold Human/Mouse/Rat). Sequencing was performed on the Illumina NextSeq 500, producing paired-end reads of 76 nucleotides in length.

### Analysis of RNA-Seq data

For RNA-Seq analysis, the raw sequencing data of the murine hippocampal samples, as well as the raw data of human tubers and controls previously published by Mills et al. 2017 (47), were imported into the Galaxy platform (66). CutAdapt (Galaxy Version 1.16.5) was used to trim reads of low sequencing quality (threshold of 20), filtering out reads with a read length less than 50 nucleotides (mouse) and human 75 nucleotides (Mills et al. 2017) (47). After read quality was ensured (FastQC; Galaxy Version 0.72+galaxy1; http://www.bioinformatics.babraham.ac.uk/projects/fastqc/), reads were mapped to the mouse reference genome GRCm38/mm10 or the human reference genome GRCh38 utilizing default settings of the STAR algorithm (Galaxy Version 2.7.2b; ref. 67). Transcript assembly was guided by the reference annotation file Gencode version M24 for mouse or version V34 for human (68). To assess counts per gene, we analyzed the mapped data sets with the featureCounts tool (Galaxy Version 1.6.4+galaxy1; ref. 69). For the PCA, normalized gene expression counts were analyzed with the DESeq2 tool (Galaxy Version 2.11.40.6+galaxy1; ref. 70). DE genes for each time point versus *Tsc1-Cre*^–^ as control were assessed: adjusted *P* < 0.05, fold change abs(log_2_FC) > 0.5. Volcano plots were generated via the Volcano Plot tool (Galaxy Version 0.0.3). The KEGG BRITE database for ion channel genes (BRITE: 04040) and the GO term action potential (GO: 0001508) were utilized as a reference for further analysis (71–73). Transcripts with an expression level higher than 10 in 50% of all samples were considered truly expressed. To generate an unsupervised clustered heatmap (complete, Euclidian, on rows and columns), the heatmap2 tool (Galaxy Version 3.0.1) was used. All data have been deposited and are accessible through the NCBI’s Gene Expression Omnibus (GEO) (74) by using the GEO number GSE164306 or by following the following link: https://www.ncbi.nlm.nih.gov/geo/query/acc.cgi?acc=GSE164306

### Statistics

Experimental groups for patch-clamp experiments consisted of at least 3 mice, and on average 3 slices of the dorsal hippocampus were used. Per slice, on average 3 cells were recorded. A maximum of 8 randomly picked cells per animal were taken into the final analysis. Each time point had its control group, except for the rapamycin-treated groups (Figure 3, B and C; and Figure 4, E and F), where 1 control group was used.

The average soma size was calculated per slice, based on 15 to 20 cells. Cells of at least 3 mice in each group were measured. The values mentioned are averages ± SEM.

Data were analyzed by using GraphPad Prism 8.0. In general, data were tested on Gaussian distribution, and based on that, groups were compared with a 2-sided parametric or nonparametric test. Excitability experiments were analyzed with a 2-way RM ANOVA with genotype or treatment condition as main effect and number of action potentials as the dependent variable. A *P* value of less than 0.05 was considered significant. A 1-way ANOVA was used to analyze soma size and DE genes over time with Dunn’s test as a post hoc test and *Tsc1-Cre*^–^ as a control group.

### Study approval

All animal experiments were conducted following the European Commission Council Directive 2010/63/EU (CCD approval AVD1010020172684) and approved by the institutional animal care and use committee of the Erasmus MC, Rotterdam, the Netherlands.

## Author contributions

LMCK conducted, acquired, and analyzed the electrophysiological data and stainings. EN analyzed the full RNA-Seq data set. IW performed and analyzed stainings. MPO acquired the RNA-Seq samples. LMCK, DCR, and YE designed the experiments and wrote the main manuscript. All authors contributed to the final version of the manuscript. LMCK was assigned as first co–first author based on being the person who held the most responsibility for the project. EN was assigned as econd co–first author based on major contributions to the manuscript: performing experiments, performing analysis, and providing scientific input.

## Supplementary Material

Supplemental data

## Version 1 12/08/2021

Electronic publication
