# Supplementary material for: Identifying the temporal electrophysiological and molecular changes that contribute to TSC-associated epileptogenesis
Source: JCI Insight. 2021 Dec 8;6(23):e150120. doi: 10.1172/jci.insight.150120 (PMC8675202; doi:10.1172/jci.insight.150120)
Supplement: Supplemental data [file jciinsight-6-150120-s109.pdf]

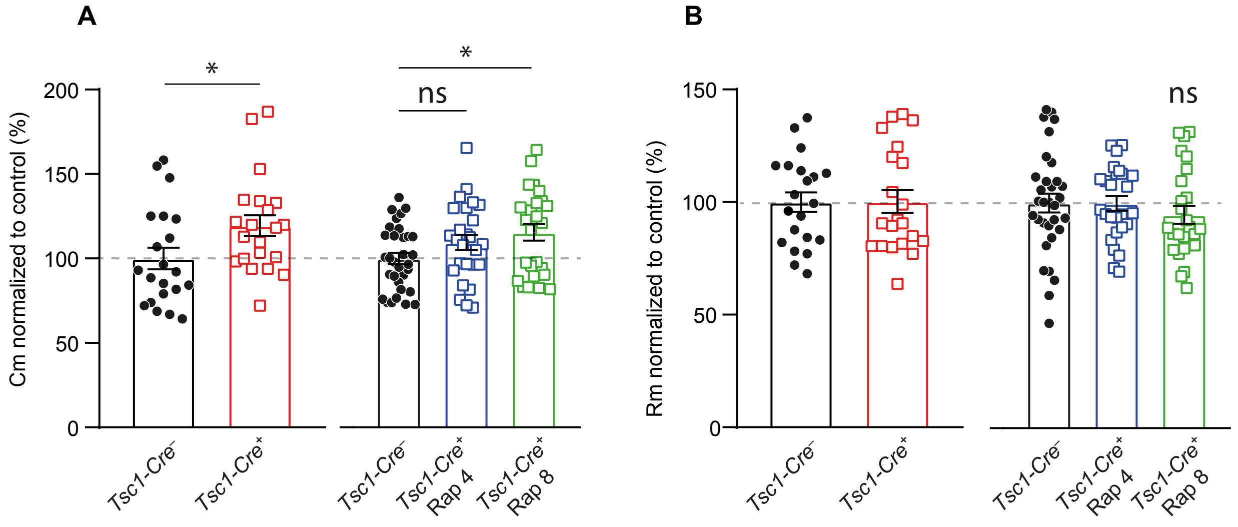

Supplementary Figure 1. Rescued cell capacitance and unaffected membrane resistance of CA1 pyramidal neurons upon early and late *in vivo* rapamycin treatment. Normalized (Cm; **A**) and membrane resistance (Rm; **B**) of CA1 pyramidal neurons of *Tsc1-Cre*⁻ (black) and *Tsc1-Cre*⁺ mice (red) recorded on day 12. Cm and Rm of the rapamycin-treated mice were normalized to their own *Tsc1-Cre*⁻ control littermates and all measured on day 12 post gene deletion (*Tsc1-Cre*⁻ n = 16, 3 mice; *Tsc1-Cre*⁺ n = 15, 3 mice; Unpaired T-test; *Tsc1-Cre*⁻ n = 30, 4 mice; vs *Tsc1-Cre*⁺-Rap: n = 26, 4 mice; and *Tsc1-Cre*⁺-Rap 8: n = 26, 4 mice. One-way ANOVA, Dunnett's with *Tsc1-Cre*⁻ as control). Error bars indicate SEM. * P<0.05.

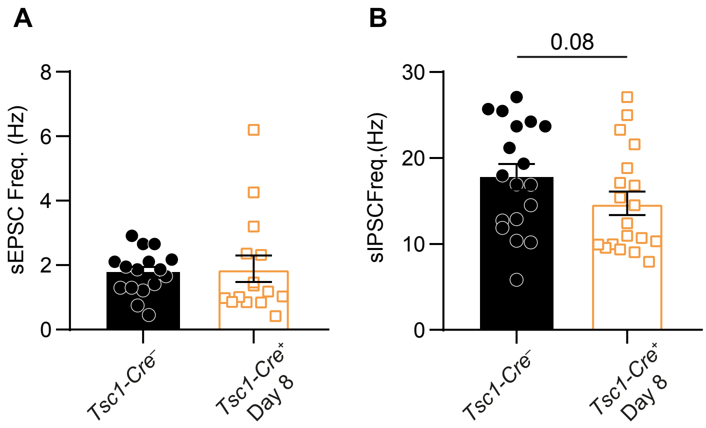

Supplementary Figure 2. Action potential dependent spontaneous excitatory and inhibitory post synaptic currents on day 8 after gene deletion. (**A**) Frequency of spontaneous excitatory post synaptic currents (sEPSC) and (**B**) spontaneous inhibitory post synaptic currents (sIPSC) measured on day 8 after *Tsc1* gene deletion. sEPSC:*Tsc1-Cre*⁻ n = 16, 3 mice; *Tsc1-Cre*⁺ n = 15, 3 mice; non-parametric Mann-Whitney U. sIPSC: *Tsc1-Cre*⁻ n = 18, 3 mice; *Tsc1-Cre*⁺ n = 19, 3 mice; non-parametric Mann-Whitney U. Error bars indicate SEM.

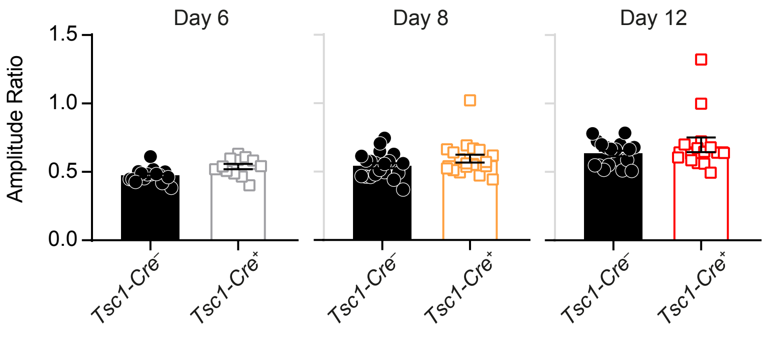

Supplementary Figure 3. Unaffected E/I amplitude ratio throughout epileptogenesis. The mEPSC/mIPSC amplitude ratio were recorded from the same cell (Day 6: *Tsc1-Cre*⁻ n = 16, 3 mice; *Tsc1-Cre*⁺ n = 12, 3 mice; independent T-test), day 8 (*Tsc1-Cre*⁻ n = 26, 4 mice; *Tsc1-Cre*⁺ n = 30, 6 mice; independent T-test) and day 12 (*Tsc1-Cre*⁻ n = 17, 4 mice; *Tsc1-Cre*⁺ n = 16, 3 mice; independent T-test). Error bars indicate SEM. v

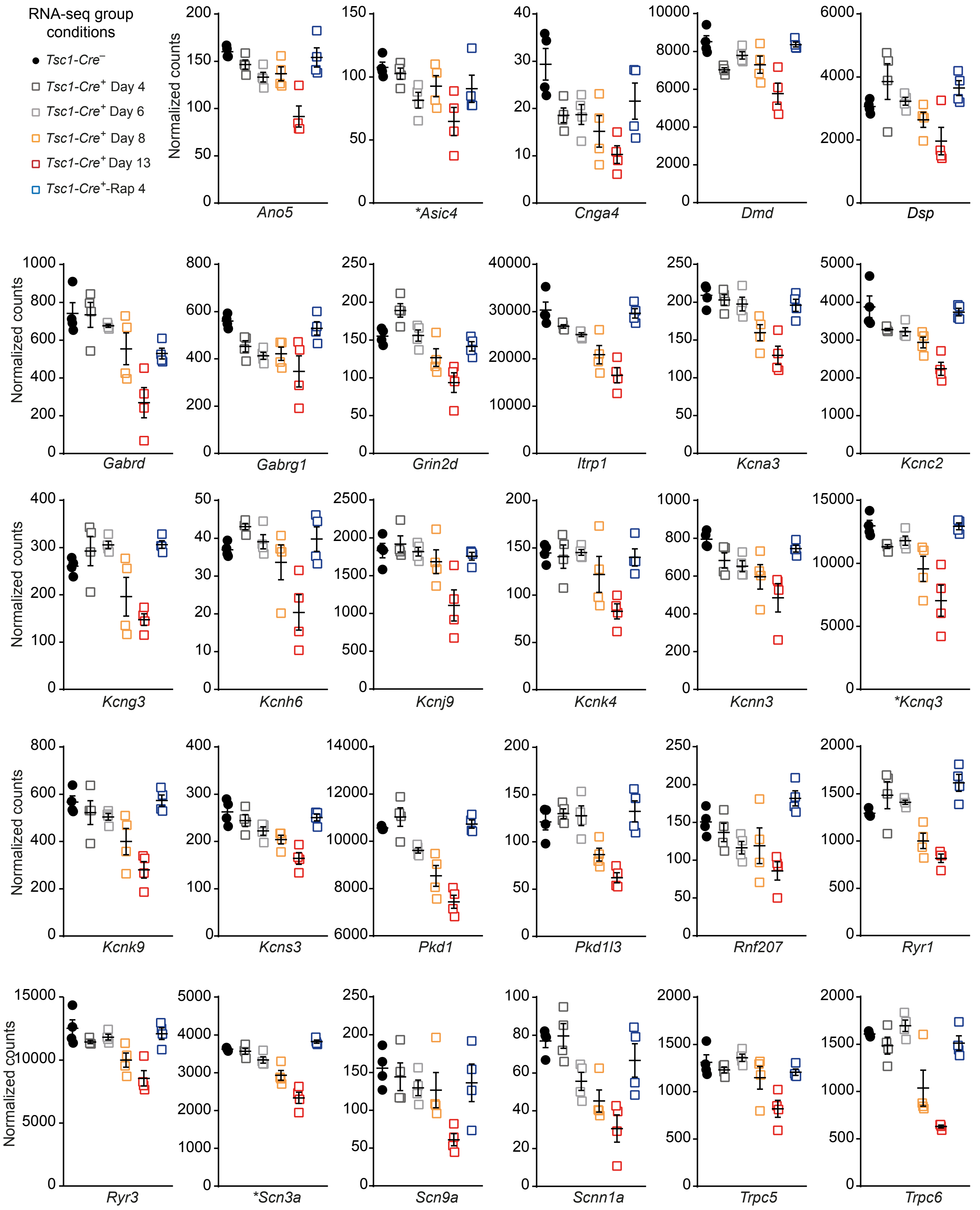

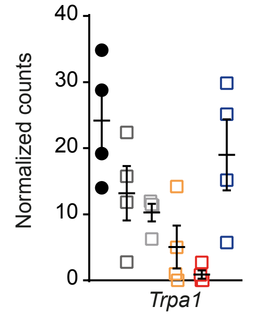

Supplementary Figure 4. Significant downregulaion of 30 transcripts during epileptogenesis of which 29 are rescued by rapamycin treatment. Panels show normalized transcript counts of hippocampal tissue of *Tsc1-Cre*−, *Tsc1-Cre*+ day 4, day 6, day 8, day 13 and *Tsc1-Cre*+-Rap (One-way ANOVA; Dunnett's with *Tsc1-Cre*− as control). Additional statistics can be found in Supplementary table 3. Genes found in TSC patient RNA-seq data are indicated by *(Mills et al. 2017).

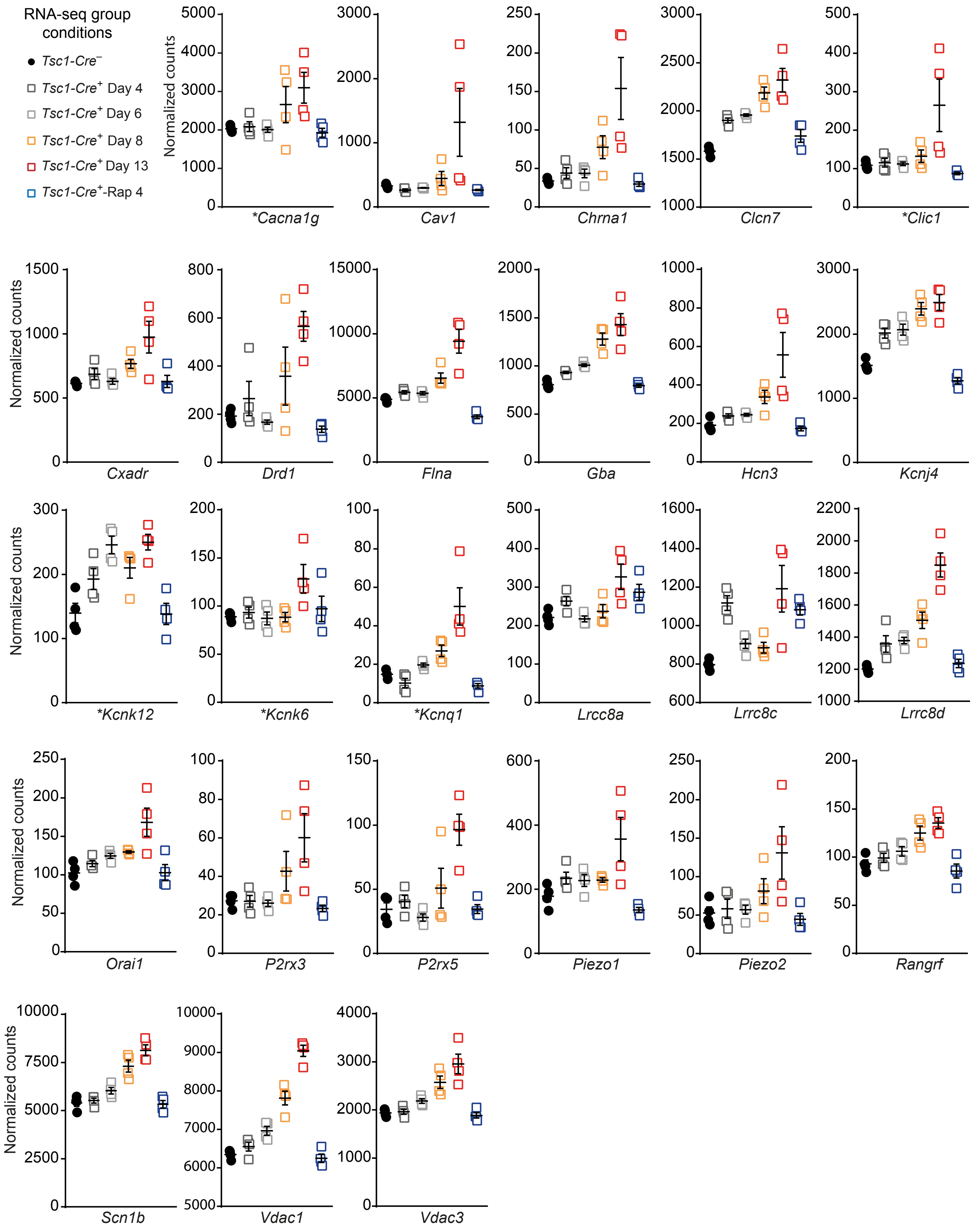

Supplementary Figure 5. Significant upregulation of 26 transcripts during epileptogenesis of which 25 are rescued by rapamycin treatment. Panels show normalized transcript counts of hippocampal tissue of *Tsc1-Cre*⁻, *Tsc1-Cre*⁺ day 4, day 6, day 8, day 13 and *Tsc1-Cre*⁺-Rap (One-way ANOVA; Dunnett's with *Tsc1-Cre*⁻ as control). Additional statistics can be found in Supplementary table 4. Genes found in TSC patient RNA-seq data are indicated by *(Mills et al. 2017).

Supplementary Table 1. Statistics of the passive and active membrane properties of *Tsc1-Cre*⁻ and *Tsc1-Cre*⁺ CA1 pyramidal neurons.

	Day 6			Day 8			Day 12		
	Tsc1-Cre⁻	*Tsc1-Cre*⁺	Statistics	*Tsc1-Cre*⁻	*Tsc1-Cre*⁺	Statistics	*Tsc1-Cre*⁻	*Tsc1-Cre*⁺	Statistics
V_{rest} (mV)	-48.29 ± 2.73	-52.06 ± 2.27	T (28) = 0.97; P = 0.34	-47.13 ± 2.48	-51.1 ± 2.045	T(34) = 1.24; P = 0.22	-50.06 ± 1.85	-48.77 ± 1.87	T(27) = 0.42; P = 0.68
Cm (pF)	111.37 ± 7.13	107.2 ±4.25	T (32) = 0.73; P = 0.47	109.5 ± 5.49	110.1 ± 2.96	T(42) = 0.11; P = 0.91	113.9 ± 7.07	137.4 ± 7.17	T(41) = 2.334; P = 0.025
Rm (MΩ)	Med: 172.5	Med: 179.5	Mann-Whitney U = 118.5; P = 0.38	178.3 ± 7.62	189.9 ± 9.64	T(42) = 0.95; P = 0.35	179.5 ± 7.48	179.4 ± 9.14	T(41) = 0.0099; P = 0.99
Rheobase (pA)	91.76 ± 10.01	85.88 ± 5.36	T(32) = 0.52; P = 0.61	Med: 80	Med: 80	Mann-Whitney U = 205.5; P = 0.38	82.33 ± 4.8	97.93 ± 5.28	T(40) = 1.62; P = 0.11
Threshold (mV)	-46.31 ± 0.44	-45.96 ± 0.97	T (32) = 0.41; P = 0.68	-44.43 ± 0.92	-45.02 ± 0.80	T (40) = 0.48; P = 0.63	-45.21 ± 0.52	-45.95 ± 0.83	T (37) = 0.78; P = 0.44
Peak Amplitude 1st AP at rheobase (mV)	Med: 97.97	Med: 97.15	Mann-Whitney U = 112; P = 0.28	98.61 ± 1.70	94.74 ± 1.12	T (40) = 0.68; P = 0.51	98.13 ± 0.59	99.71 ± 1.11	T (37) = 1.3; P = 0.19
10-90 % rise time (mV/ms)	334.59 ± 9.23	341.81 ± 7.4	T (32) = 0.61; P = 0.54	389.8 ± 18.66	383.80 ± 13.41	T (40) = 0.266; P = 0.79	334.56 ± 6.8	345.43 ± 9.17	T (37) = 0.96; P = 0.34
10-90 % decay time (mV/ms)	-76.89 ± 1.7	-79.57 ± 1.73	T (32) = 1.09; P = 0.28	-66.68 ± 2.36	-70.51 ± 1.82	T (40) = 1.31; P = 0.197	-75.702 ± 1.65	-78.61 ± 1.61	T (37) = 1.25; P = 0.22
Half-width (ms)	1.08 ± 0.017	1.08 ± 0.013	T (32) = 0.12; P = 0.90	1.27 ± 0.04	1.243± 0.02	T (40) = 0.92; P = 0.36	1.11 ± 0.023	1.18 ± 0.025	T (37) = 2.31; P = 0.026
Ih (%)	6.68 ± 0.28	5.88 ± 0.4	T (32) = 1.58; P = 0.122	6.10 ± 0.32	6.29 ± 0.31	T (37) = 0.39; P = 0.69	6.58 ± 0.34	7.41 ± 0.51	T (37) = 1.37; P = 0.18
Latency (ms)	22.61 ± 4.52	33.36 ± 3.21	T(32) = 1.97; P = 0.06	51.31 ± 3.93	55.86 ± 3.06	T(40) = 0.93; P = 0.36	30.86 ± 4.05	34.09 ± 3.98	T(37) = 0.563; P = 0.58

Supplementary Table 2. Statistics of mEPSC and mIPSC kinetics of CA1 pyramidal neurons.

		Day 6			Day 8			Day 12		
		Tsc1-Cre⁻	*Tsc1-Cre⁺*	Statistics	*Tsc1-Cre⁻*	*Tsc1-Cre⁺*	Statistics	*Tsc1-Cre⁻*	*Tsc1-Cre⁺*	Statistics
**mEPSC properties**	Frequency (Hz)	0.75 ± 0.077	0.6 ± 0.096	T(39) = 0.21<br>P = .22	0.51 ± 0.052	0.82 ± 0.14	T(54) = 1.89<br>P =0 .06	0.56 ± 0.053	0.46 ± 0.054	T(34) =1.30<br>P = 0.20
	Amplitude (pA)	-9.65 ± 0.25	-9.67 ± 0.29	T(34) = 0.07;<br>P = 0.94	-11.21 ± 0.27	-11.06 ± 0.43	T(36) = 0.29;<br>P = .78	Med:<br>-9.15	Med:<br>-8.36	Mann-Whitney<br>U = 84.0; P =0 .0047
	Rise time (10-90% ms)	Med: 1.46	Med: 1.67	Mann-Whitney<br>U = 88.0; P =<br>0.025	1.61 ± 0.41	1.79 ± 0.58	T(36) = 1.15;<br>P = 0.25	1.66 ± 0.09	1.78 ± 0.08	T(36) = 1.02;<br>P = 0.32
	Decay time constant (10-90% ms)	6.79 ± 0.34	6.95 ± 1.25	T(34) = 0.346;<br>P = 0.74	7.51 ± 0.23	7.6 ± 0.34	T(36) = 0.20;<br>P = 0.84	7.98 ± 0.33	6.71 ± 0.26	T(36) = 2.91;<br>P = 0.0061
**mIPSC properties**	Frequency (Hz)	5.69 ± 0.45	5.88 ± 0.46	T(26) = 0.77<br>P = 0.77	5.47 ± 0.40	4.82 ± 0.29	T(44) = 1.35<br>P = 0.18	4.52 ± 0.34	2.31 ± 0.31	T(29) = 4.72<br>P = 0.0001
	Amplitude (pA)	20.42 ± 0.58	19.06 ± 0.68	T(25) = 1.53;<br>P = 0.14	19.45 ± 0.58	18.57 ± 0.58	T(33) = 1.06;<br>P = 0.29	15.27 ± 0.39	13.16 ± 0.38	T(31) = 3.82;<br>P = 0.0006
	Rise time (10-90% ms)	0.55 ± 0.024	0.61 ± 0.028	T(25) = 1.78;<br>P = 0.09	0.58 ± 0.03	0.66 ± 0.03	T(33) = 1.79;<br>P = 0.082	0.53 ± 0.02	0.75 ± 0.04	T (31) = 4.98;<br>P = 0.0001
	Decay time constant (10-90% ms)	11.85 ± 1.25	12.91 ± 1.31	T(25) = 0.57;<br>P = 0.57	14.08 ± 1.171	16.39 ± 1.3	T(33) = 1.29;<br>P = 0.2	13.57 ± 1.23	13.15 ± 1.28	T (31) = 0.23;<br>P = 0.81

Supplementary Table 3. Detailed statistics of downregulated DE ‘action potential’ genes overtime.

				Tsc1-Cre^{-}		*Tsc1-Cre*$^{+}$-Day 4			*Tsc1-Cre*$^{+}$-Day 6			*Tsc1-Cre*$^{+}$-Day 8			*Tsc1-Cre*$^{+}$-Day 13			*Tsc1-Cre*$^{+}$-Rap 4		
	**GeneID**	**Gene**	**One-way ANOVA (Dunnet’s post hoc)**	**Mean**	**SEM**	**Mean**	**SEM**	**Sig**	**Mean**	**SEM**	**Sig**	**Mean**	**SEM**	**Sig**	**Mean**	**SEM**	**Sig**	**Mean**	**SEM**	**Sig**
1	ENSMUSG 00000055489	*Ano5*	F(5, 18) = 10.65<br>P < 0.0001	160.10	2.98	146.62	4.76	0.59	133.26	5.12	0.07	136.86	7.33	0.14	91.61	11.07	0.0001	154.04	10.14	0.96
2	ENSMUSG 00000033007	**Asic4*	F(5, 18) = 3.68<br>P = 0.0180	107.67	4.09	102.61	4.40	0.98	81.50	6.25	0.12	92.63	8.37	0.56	64.68	11.18	0.0059	90.64	10.86	0.45
3	ENSMUSG 00000030897	*Cnga4*	F(5, 18) = 5.21<br>P = 0.0039	29.38	3.33	18.56	1.55	0.054	18.74	2.11	0.059	15.18	3.35	0.0091	10.28	1.87	0.0006	21.58	3.82	0.22
4	ENSMUSG 00000045103	*Dmd*	F(5, 18) = 8.99<br>P = 0.0002	8514.04	321.34	7027.31	195.35	0.02	7792.36	195.68	0.44	7295.8	453.93	0.07	5763.88	556.28	0.0001	8362.87	125.34	0.99
5	ENSMUSG 00000054889	*Dsp*	F(5, 18) = 4.41<br>P = 0.008	3063.25	101.93	3855.32	564.98	0.33	3227.30	123.76	0.99	2640.59	242.46	0.82	1963.39	434.10	0.1	3646.74	236.32	0.6
6	ENSMUSG 00000029054	*Gabrd*<br>(GABAδ)	F(5, 18) = 8.63<br>P = 0.0003	741.48	57.51	733.67	66.17	0.99	675.78	7.62	0.90	554.32	84.23	0.15	269.23	80.07	0.0002	529.08	28.23	0.08
7	ENSMUSG 00000001260	*Gabrg1*<br>(GABAγ1)	F(5, 18) = 5.42<br>P = 0.003	559.91	13.86	453.22	23.78	0.13	413.22	14.84	0.027	421.94	27.96	0.039	347.15	65.67	0.0014	529.34	28.18	0.94
8	ENSMUSG 00000002771	*Grin2d*<br>(NMDA2D)	F(5, 18) = 12.17<br>P < 0.0001	155.13	5.17	189.14	9.00	0.06	155.92	7.43	0.99	126.71	11.62	0.15	93.69	13.04	0.0008	141.65	6.22	0.75
9	ENSMUSG 00000030102	*Itpr1*	F(5, 18) = 16.19<br>P < 0.0001	30329.09	1696.68	26877.54	374.44	0.26	25134.55	419.17	0.04	20907.05	1935.79	0.0004	16556.27	1593.88	0.0001	29643.23	1002.85	0.99
10	ENSMUSG 00000047959	*Kcna3*<br>(Kv1.3)	F(5, 18) = 11.38<br>P < 0.0001	208.86	7.28	203.02	6.97	0.98	197.49	8.96	0.84	159.65	10.73	0.0006	129.67	12.12	0.0001	195.86	8.11	0.77
11	ENSMUSG 00000035681	*Kcnc2*<br>(Kv3.2)	F(5, 18) = 13.63<br>P < 0.0001	3879.38	287.63	3276.40	31.48	0.061	3222.38	105.52	0.038	2935.95	143.54	0.002	2239.89	169.99	0.0001	3739.73	98.43	0.96
12	ENSMUSG 00000045053	*Kcng3*<br>(Kv6.3)	F(5, 18) = 8.62<br>P = 0.0003	260.73	8.51	292.83	31.10	0.76	305.60	8.17	0.49	196.21	41.01	0.19	147.60	12.38	0.009	306.39	8.03	0.48
13	ENSMUSG 00000001901	*Kcnh6*<br>(Kv11.2)	F(5, 18) = 6.63<br>P = 0.001	36.98	0.94	43.09	0.84	0.51	39.09	1.89	0.98	33.62	4.58	0.9	20.38	4.68	0.006	39.77	3.23	0.95
14	ENSMUSG 00000038026	*Kcnj9*<br>(Kir3.3)	F(5, 18) = 5.56<br>P = 0.0029	1831.54	96.41	1917.66	107.19	0.98	1817.90	57.07	0.99	1685.28	157.04	0.87	1105.95	205.97	0.002	1756.87	50.24	0.99
15	ENSMUSG 00000024957	*Kcnk4*<br>(TRAAK)	F(5, 18) = 5.10<br>P = 0.0044	144.77	4.81	140.84	12.32	0.99	145.24	3.08	0.99	121.99	19.00	0.44	82.91	8.06	0.003	139.97	9.09	0.99
**16**	ENSMUSG 00000036760	*Kcnk9*<br>(TASK3)	F(5, 18) = 9.37<br>P = 0.0002	567.46	25.65	522.79	50.46	0.86	504.42	14.10	0.64	400.07	55.61	0.021	280.46	35.23	0.0002	573.57	23.93	0.99
17	ENSMUSG 00000000794	*Kcnn3*<br>(SK3)	F(5, 18) = 5.60<br>P = 0.002	794.42	21.07	682.28	39.94	0.33	651.80	28.90	0.15	595.73	64.41	0.029	484.91	75.16	0.0008	745.06	17.77	0.91
18	ENSMUSG 00000056258	**Kcnq3*<br>(Kv7.3)	F(5, 18) = 10.70<br>P < 0.0001	12972.57	431.86	11324.96	152.11	0.35	11792.91	358.06	0.64	9553.82	997.13	0.017	7042.54	1234.75	0.0001	12938.20	269.68	0.99
19	ENSMUSG 00000043673	*Kcns3*<br>(Kv9.3)	F(5, 18) = 11.33<br>P < 0.0001	262.60	13.25	244.32	12.42	0.64	222.33	9.01	0.064	204.20	9.23	0.005	164.23	12.24	0.0001	250.82	7.20	0.89
20	ENSMUSG 00000032855	*Pkd1*	F(5, 18) = 25.54<br>P < 0.0001	10564.87	36.74	11028.39	393.84	0.66	9615.08	115.35	0.1	8538.27	439.17	0.0003	7441.26	278.32	0.0001	10728.32	174.77	0.99
21	ENSMUSG 00000048827	*Pkd1l3*	F(5, 18) = 12.31<br>P < 0.0001	121.20	8.46	129.85	5.22	0.91	127.70	10.42	0.97	86.56	6.87	0.03	62.05	5.31	0.0004	132.25	11.15	0.86

22	ENSMUSG 00000058498	**Rnf207*	F(5, 18) = 5.92 P = 0.002	150.62	8.58	136.75	12.07	0.92	116.48	23.65	0.29	119.09	23.65	0.36	85.71	12.11	0.013	181.85	9.94	0.37
23	ENSMUSG 00000030592	*Ryr1*	F(5, 18) = 14.70 P < 0.0001	1293.03	19.72	1485.25	142.57	0.32	1409.94	21.38	0.74	1001.65	81.12	0.069	818.23	44.66	0.002	1616.77	89.63	0.039
**24**	ENSMUSG 00000057378	*Ryr3*	F(5, 18) = 9.72 P = 0.0001	12517.11	667.81	11432.48	121.14	0.39	11816.40	225.41	0.75	9993.19	562.78	0.007	8552.84	600.93	0.0001	12092.26	456.70	0.95
25	**ENSMUSG 00000057182**	***Scn3a* (Nav1.3 α-subunit)**	**F(5, 18) = 33.98 P < 0.0001**	**3620.85**	**19.05**	**3567.24**	**75.04**	**0.99**	**3339.37**	**85.39**	**0.17**	**2931.03**	**130.10**	**0.0003**	**2335.78**	**146.38**	**0.0001**	**3823.54**	**41.15**	**0.43**
26	ENSMUSG 00000075316	*Scn9a*	F(5, 18) = 3.64 P = 0.0189	155.65	12.64	144.30	18.25	0.98	129.71	10.29	0.74	126.76	23.31	0.66	61.13	8.09	0.005	136.37	24.92	0.89
27	ENSMUSG 00000030340	*Scnn1a*	F(5, 18) = 9.04 P = 0.0002	76.91	3.43	79.58	6.54	0.99	55.58	4.75	0.10	45.16	5.82	0.0097	30.61	7.19	0.0003	66.64	8.82	0.67
28	ENSMUSG 00000041710	*Trpc5*	F(5, 18) = 6.88 P = 0.0009	1311.73	78.23	1231.29	30.47	0.89	1359.62	38.23	0.98	1147.94	120.37	0.39	819.69	88.93	0.0007	1207.34	33.77	0.76
29	ENSMUSG 00000031997	*Trpc6*	F(5, 18) = 18.71 P < 0.0001	1609.29	12.99	1485.21	89.65	0.82	1695.72	60.83	0.94	1036.36	189.68	0.002	630.99	13.84	0.0001	1512.63	77.88	0.92
30	ENSMUSG 00000032769	*Trpa1*	F(5, 18) = 5.59 P = 0.0028	24.21	4.67	13.20	4.10	0.16	10.27	1.34	0.057	5.06	3.24	0.0069	0.89	0.65	0.0012	18.99	5.37	0.76

Supplementary Table 4. Detailed statistics of upregulated DE 'action potential' genes overtime.

				Tsc1-Cre^{-}		*Tsc1-Cre*$^{+}$-Day 4			*Tsc1-Cre*$^{+}$-Day 6			*Tsc1-Cre*$^{+}$-Day 8			*Tsc1-Cre*$^{+}$-Day 13			*Tsc1-Cre*$^{+}$-Rap 4		
	**GeneID**	**Gene**	**One-way ANOVA (Dunnet's post hoc)**	**Mean**	**SEM**	**Mean**	**SEM**	**Sig**	**Mean**	**SEM**	**Sig**	**Mean**	**SEM**	**Sig**	**Mean**	**SEM**	**Sig**	**Mean**	**SEM**	**Sig**
1	ENSMUSG 00000020866	**Cacna1g*	F(5, 18) = 3.21<br>P = 0.0302	2032.02	40.48	2085.58	124.97	0.99	2006.47	68.51	0.99	2657.52	467.56	0.34	3095.98	397.22	0.04	1930.36	115.20	0.99
2	ENSMUSG 00000007655	*Cav1*	F(5, 18) = 22.63<br>P = 0.02	330.3	15.64	263.4	17.38	0.99	287.5	6.22	0.99	444.7	107.8	0.99	1319	528.9	0.022	264	9.61	0.99
3	ENSMUSG 00000027107	*Chrna1*	F(5, 18) = 6.93<br>P = 0.0009	33.61	1.68	43.98	7.02	0.99	43.65	5.56	0.99	77.71	14.86	0.32	153.87	40.30	0.0008	29.66	2.89	0.99
4	ENSMUSG 00000036636	*Clcn7*	F(5, 18) = 18.47<br>P < 0.0001	1582.31	23.93	1900.81	25.10	0.009	1958.03	12.02	0.0025	2187.02	61.79	0.0001	2320.47	122.01	0.0001	1740.86	65.59	0.31
5	ENSMUSG 00000007041	**Clic1*	F(5, 18) = 4.89<br>P = 0.0053	108.76	5.01	115.62	11.44	0.99	112.41	4.46	0.99	132.55	15.82	0.96	264.65	67.93	0.006	87.56	3.04	0.98
6	ENSMUSG 00000022865	*Cxadr*	F(5, 18) = 5.46<br>P = 0.0031	613.55	8.41	686.24	42.45	0.85	629.46	21.75	0.99	766.95	35.3	0.27	973.92	123.27	0.0019	629.2	46.95	0.99
7	ENSMUSG 00000021478	*Drd1*	F(5, 18) = 6.45<br>P = 0.0013	192.06	13.44	265.31	71.29	0.87	166.99	8.82	0.99	358.08	120.38	0.26	565.06	62.32	0.002	137.64	12.91	0.95
**8**	**ENSMUSG 00000031328**	***Flna***	**F5, 18) = 22.34<br>P < 0.0001**	**4889.15**	**88.28**	**5441.7**	**121.22**	**0.82**	**5361.06**	**124.155**	**0.89**	**6547.12**	**406.04**	**0.048**	**9417.26**	**918.61**	**0.0001**	**3532.96**	**150.75**	**0.13**
9	ENSMUSG 00000028048	*Gba*	F(5, 18) = 22.48<br>P < 0.0001	806.10	20.25	930.54	9.75	0.38	1008.1	13.6	0.06	1280.5	62.72	0.0001	1429.8	114.61	0.0001	794.27	16.68	0.99
10	ENSMUSG 00000028051	*Hcn3*	F(5, 18) = 8.02<br>P = 0.0004	190.42	15.97	239.16	10.55	0.93	245.33	6.78	0.89	337.75	34.60	0.18	556.26	115.90	0.0003	173.14	11.16	0.99
11	ENSMUSG 00000044216	*Kcnj4* (Kir2.3)	F(5, 18) = 32.54<br>P < 0.0001	1511.66	42.21	2016.42	79.21	0.0022	2070.06	85.74	0.0009	2395.53	97.97	0.0001	2494.50	123.75	0.0001	1274.66	50.12	0.21
**12**	**ENSMUSG 00000050138**	***Kcnk12* (2PK channel)**	**F(5. 18) = 10.74<br>P < 0.0001**	**139.50**	**15.23**	**192.84**	**16.21**	**0.08**	**245.89**	**13.53**	**0.0004**	**210.17**	**16.17**	**0.016**	**250.23**	**12.13**	**0.0003**	**138.26**	**16.51**	**0.99**
13	ENSMUSG 00000046410	**Kcnk6* (TWIK2, TOSS)	F(5, 18) = 2.94<br>P = 0.0409	88.98	2.23	93.27	5.64	0.99	87.18	6.60	0.99	88.54	4.81	0.99	128.26	14.89	0.02	97.15	13.16	0.95
14	ENSMUSG 00000009545	**Kcnq1* (Kv7.1)	F(5, 18) = 12.77<br>P < 0.0001	14.87	1.05	10.24	2.40	0.9	19.67	0.94	0.89	26.98	2.95	0.21	50.04	9.65	0.0001	8.80	1.16	0.77
15	ENSMUSG 00000007476	*Lrrc8a*	F(5, 18) = 5.18<br>P = 0.0041	221.02	9.07	263.56	12.18	0.38	217.43	7.50	0.99	237.11	17.28	0.96	326.72	32.88	0.0036	286.55	20.55	0.086
16	ENSMUSG 00000054720	*Lrrc8c*	F(5, 18) = 5.95<br>P = 0.002	796.38	13.76	1117.23	37.96	0.002	905.18	24.28	0.51	884.602	27.77	0.69	1191.66	120.95	0.0004	1081.92	26.9	0.007
17	ENSMUSG 00000046079	*Lrrc8d* (VRACs)	F(5. 18) = 27.77<br>P < 0.0001	1201.48	10.76	1357.44	51.09	0.09	1378.90	19.79	0.046	1505.41	50.89	0.0007	1849.74	75.18	0.0001	1236.02	26.52	0.97
18	ENSMUSG 00000049686	*Orai1*	F(5, 18) = 6.93<br>P = 0.0009	102.48	6.81	114.49	3.96	0.90	124.69	3.31	0.43	129.64	1.49	0.24	168.28	18.28	0.0005	102.84	10.43	0.99
19	ENSMUSG 00000027071	*P2rx3*	F(5, 18) = 4.40<br>P = 0.0086	27.28	1.73	27.04	3.06	0.99	26.04	1.65	0.99	42.66	10.26	0.39	60.09	12.51	0.013	23.33	1.63	0.99
20	ENSMUSG 00000005950	*P2rx5*	F(5, 18) = 8.37<br>P = 0.0003	34.48	5.09	40.48	4.85	0.98	28.00	2.80	0.98	50.90	15.51	0.55	96.48	12.06	0.0004	34.56	3.49	0.99
21	ENSMUSG 00000014444	*Piezo1*	F(5, 18) = 5.79<br>P = 0.0023	178.45	17.64	234.35	18.60	0.58	227.05	18.53	0.69	228.79	6.67	0.67	356.22	67.72	0.003	135.59	7.72	0.78

22	ENSMUSG 00000041482	*Piezo2*	F(5, 18) = 3.52 P = 0.0216	52.75	8.04	58.30	12.30	0.99	57.11	5.83	0.99	81.16	16.30	0.65	130.81	33.98	0.01	44.57	7.67	0.99
**23**	ENSMUSG 00000032892	*Rangrf*	F5, 18) = 11.04 P <0.0001	93.01	4.19	99.26	4.66	0.90	106.11	5.0	0.38	125.07	7.27	0.0044	135.36	7.26	0.0003	85.48	7.26	0.82
24	**ENSMUSG 00000019194**	***Scn1b* (Nav1.1 β-subunit)**	**F(5, 18) = 28.35 P < 0.0001**	**5403.76**	**174.34**	**5522.19**	**128.05**	**0.99**	**6030.54**	**168.23**	**0.19**	**7310.30**	**306.87**	**0.0001**	**8132.58**	**281.72**	**0.0001**	**5332.62**	**197.50**	**0.99**
25	ENSMUSG 00000020402	*Vdac1*	F(5, 18) = 74.93 P < 0.0001	6347.18	57.68	6553.16	113.92	0.67	6965.88	115.99	0.01	7814.14	177.58	0.0001	9039.30	145.04	0.0001	6249.80	106.90	0.97
26	ENSMUSG 00000008892	*Vdac3*	F(5, 18) = 16.28 P < 0.0001	1938.86	37.80	1962.13	51.54	0.99	2187.14	48.93	0.35	2575.43	128.77	0.002	2954.60	203.07	0.0001	1888.15	57.47	0.99
